# Supplementary figures and images for: Excitatory and inhibitory effects of HCN channel modulation on excitability of layer V pyramidal cells
Source: PLoS Comput Biol. 2022 Sep 13;18(9):e1010506. doi: 10.1371/journal.pcbi.1010506 (PMC9506642; doi:10.1371/journal.pcbi.1010506)

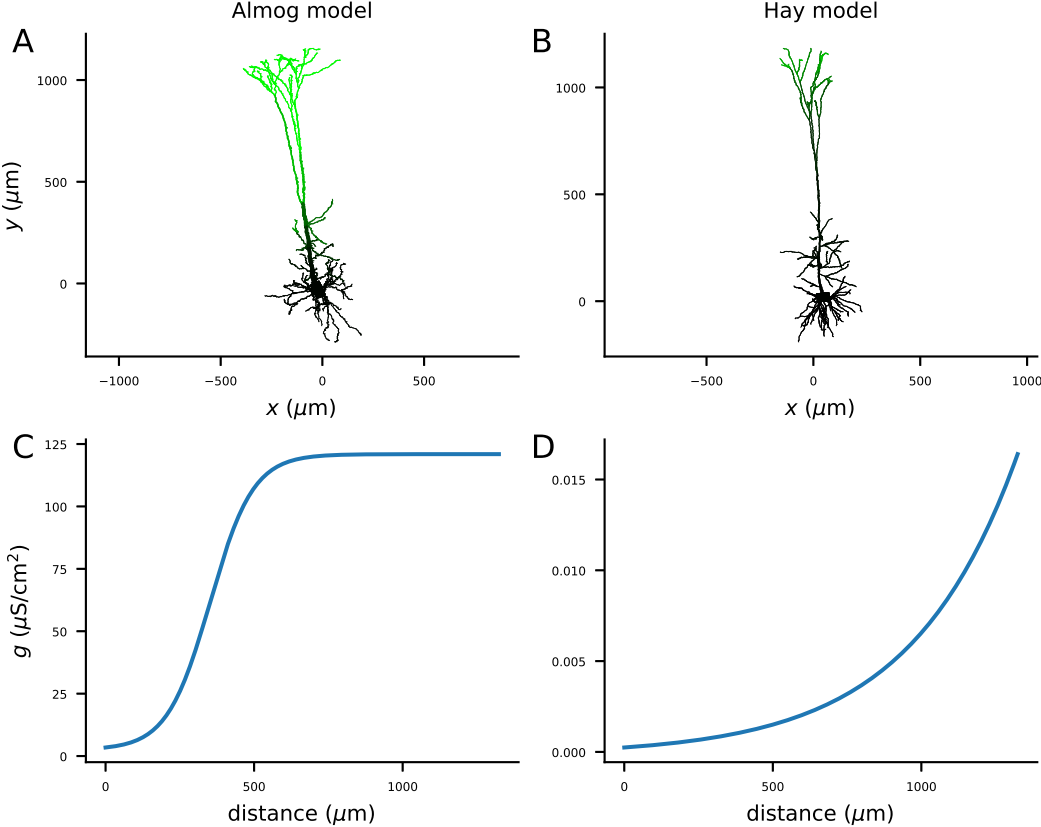

Supplement: S1 Fig — A–B: Illustration of the Ih channel conductance along the dendritic tree in Almog (A) and Hay (B) models. Black compartments indicate low Ih conductance and green compartments indicate high Ih conductance—see panels (C) and (D) for absolute values. C–D: The Ih channel conductance along the apical dendrite with respect to the distance from the soma in Almog (C) and Hay (D) models. (PDF) [file pcbi.1010506.s001.pdf]

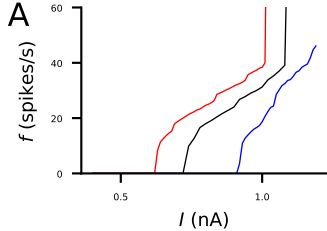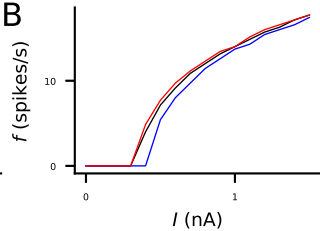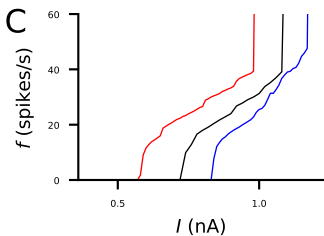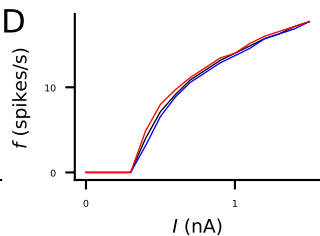

**E**

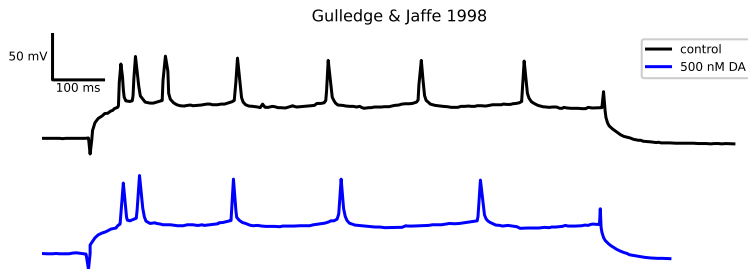

**F**

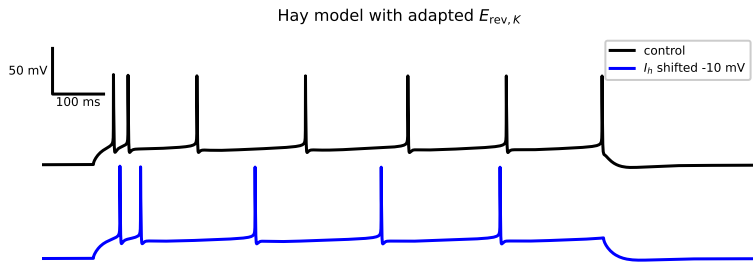

Supplement: S2 Fig — A–B: The frequency of APs (y-axis) in response to somatic DC of a given amplitude (x-axis) in Almog (A) and Hay (B) model neurons under up- or down-regulated Ih channels. Black: control neuron. Blue: Ih conductance blocked in the apical dendrite. Red: Ih conductance increased by 100% in the apical dendrite. C–D: The frequency of APs in response to somatic DC in Almog (C) and Hay (D) model neurons under different neuromodulatory states. Black: control neuron. Blue: cAMP-inhibiting neuromodulation. Red: cAMP-enhancing neuromodulation. E: Membrane potential time course data from [3] measured from a control L5PC (black) and an L5PC when bath-applied with 500 nM dopamine (blue). Data digitized from Figure 6A of [3]. F: Somatic membrane potential time course predicted by the Hay model for a 0.44-nA somatic stimulation of 1 second in control L5PC (black) and under cAMP-inhibiting neuromodulation (blue). The reversal potential was adapted to -88.4 mV (originally -85 mV in the Hay model) to account for the differences in intracellular K+ concentrations (140 mM in [3], 120 mM in the experiments underlying the Hay model). The shift of -10 mV in the half-inactivation voltage of Ih in the Hay model had the same effect on the number of APs in response to 1-second stimulus (decreased from 7 to 5 APs) as the bath-application of 500 nM dopamine in [3]. (PDF) [file pcbi.1010506.s002.pdf]

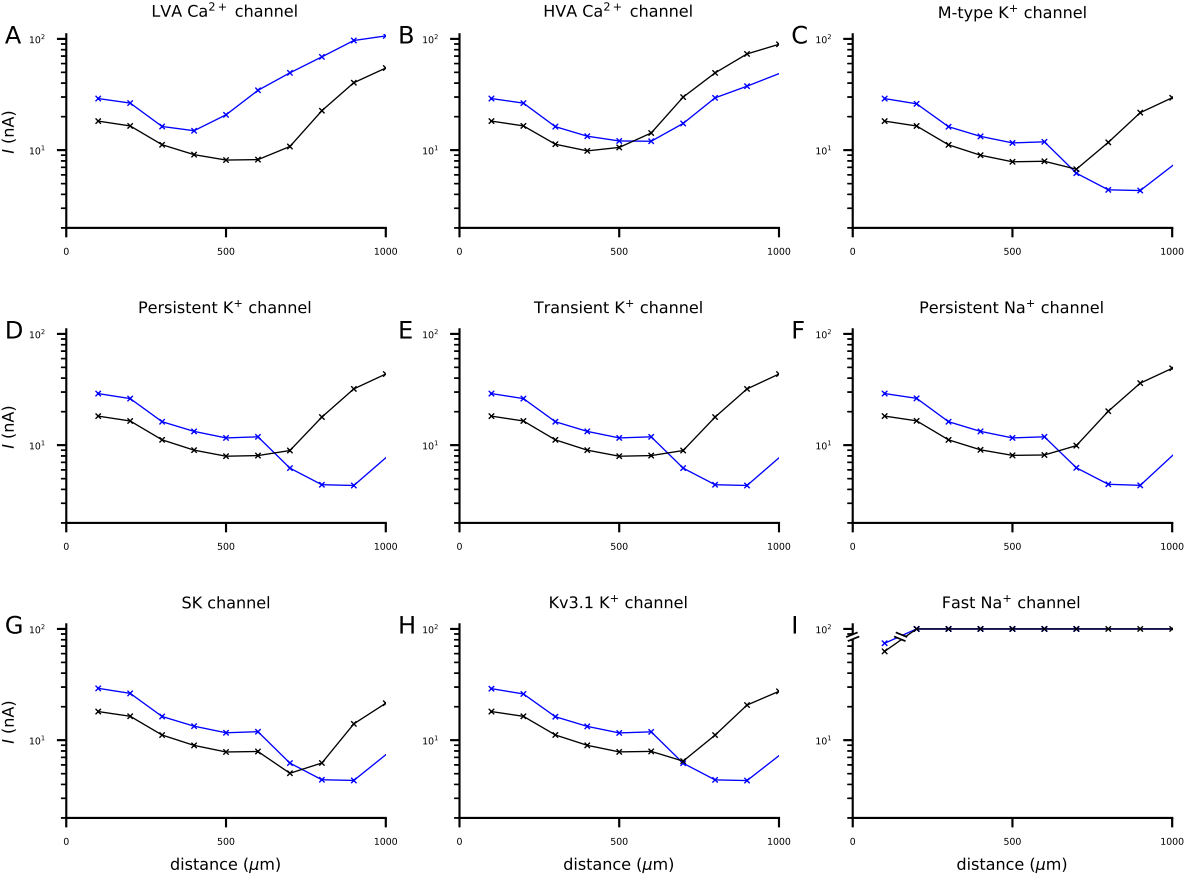

Supplement: S3 Fig — See Fig 2 for details. A: LVA Ca2+ channels blocked. B: HVA Ca2+ channels blocked. C: M-type K+ channels blocked. D: Persistent K+ channels blocked. E: Transient K+ channels blocked. F: Persistent Na+ channels blocked. G: Ca2+-dependent K+ channels (SK channels) blocked. H: Kv3.1-type K+ channels blocked. I: Transient Na+ channels blocked. Black curves: the named ion channel blocked. Blue curves: the named ion channel and the Ih channel blocked. (PDF) [file pcbi.1010506.s003.pdf]

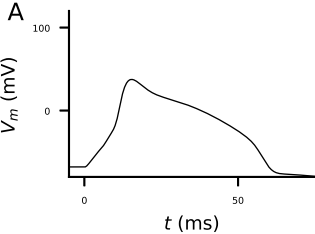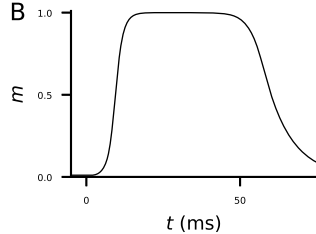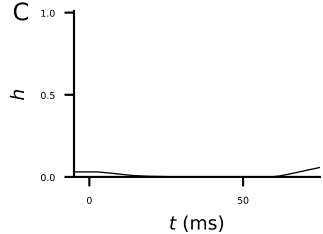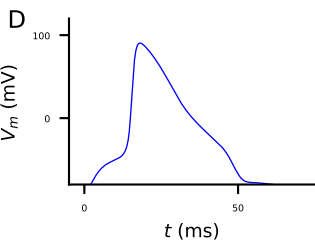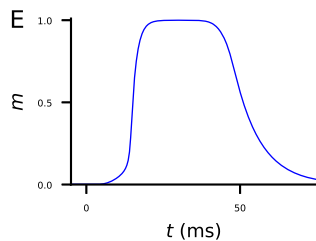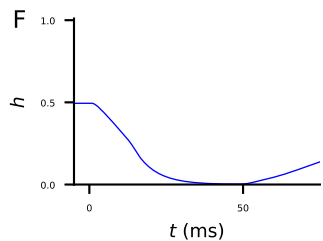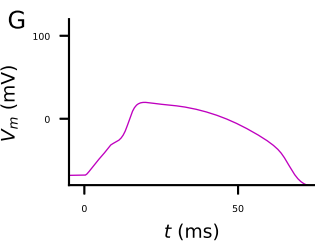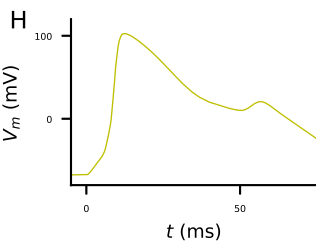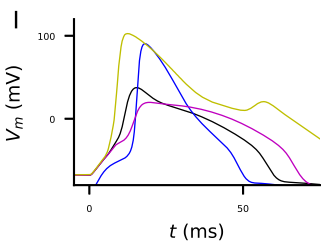

Supplement: S4 Fig — A–C: Time courses of the membrane potential (A) and the activation (B) and inactivation (C) variables m and h (Eqs 3 and 4) of the LVA Ca2+ channels in the control condition when the compartment was stimulated with an alpha-shaped synaptic conductance of 2 nS. D–F: Time courses of the membrane potential (D) and the activation (E) and inactivation (F) variables m and h when Ih channels were blocked. Note the significantly larger values of h in (F) compared to control (C). G: Time course of the membrane potential of a model compartment, where the LVA Ca2+ current is replaced by an artificial LVA current species where the values of the activation variable m (Eq 3) are directly taken from Ih-blocked simulation (E) and those of the inactivation variable h (Eq 4) are taken from the control simulation (C). This model compartment produces a milder response than either the control (A) or Ih-blocked neuron (D). H: Time course of the membrane potential of a model compartment, where the LVA Ca2+ current is replaced by an artificial LVA current species where activation variable m is taken from the control simulation (B) and the inactivation variable h is taken from the Ih-blocked simulation (F). This model compartment produces a stronger response than either the control (A) or Ih-blocked neuron (D). I: The membrane potential time courses from panels (A), (D), and (G–H) overlaid. The observation that the response in (H) reached (and went beyond) that of the Ih blocked neuron indicates that increase in dendritic spike magnitude caused by Ih blockage is due to altered levels of inactivation, not activation, of LVA Ca2+ channels. (PDF) [file pcbi.1010506.s004.pdf]

—  $V_{\text{soma}}$  —  $V_{\text{dend}}$

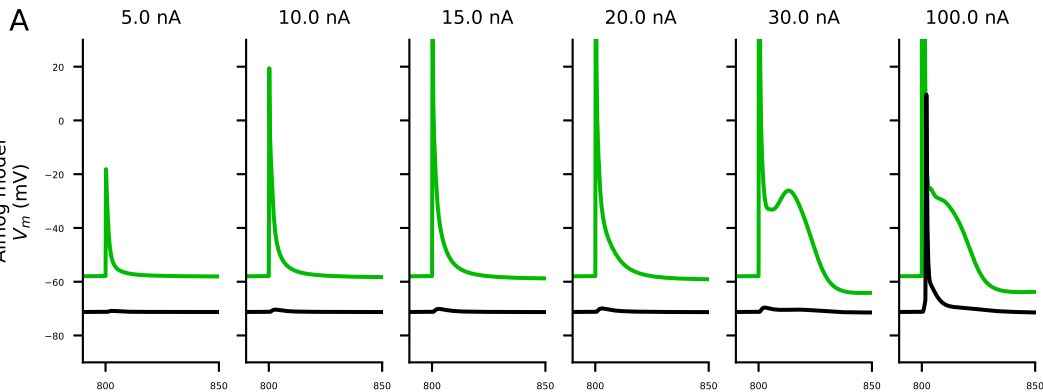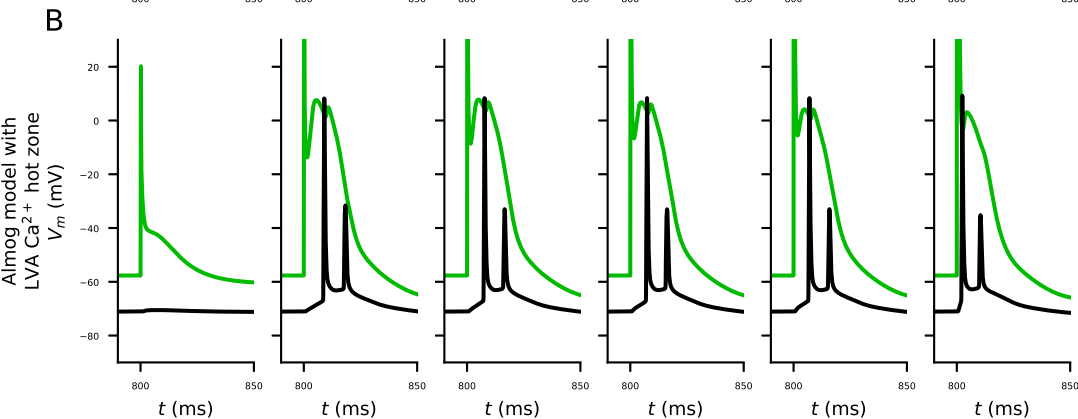

Supplement: S5 Fig — A–B: Time courses of the membrane potential of the original Almog model (A) and the Almog model with a hot zone (B) in response to short (0.2 ms) apical dendritic square-pulse current at 800 μm from the soma, recorded at the location of input (green) and at soma (black). The stimulus intensity was varied from 5.0 nA (left) to 100 nA (right). The Almog model with a hot zone of LVA Ca2+ produces APs for smaller stimulus intensity and exhibits a stronger dendritic spike that leads to a burst of somatic APs instead of a single spike. (PDF) [file pcbi.1010506.s005.pdf]

Almog with hot zone, cAMP-enhanced vs. control

Almog with hot zone, cAMP-inhibited vs. control

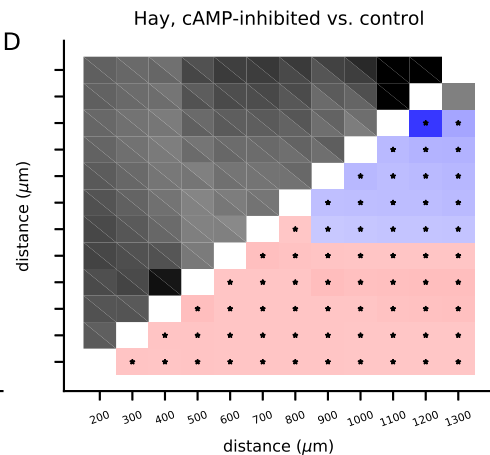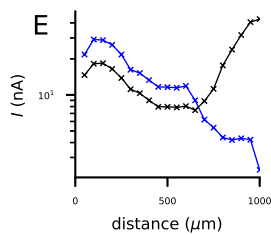

Supplement: S8 Fig — A–D: Effects of weak Ih channel modulation on apical dendritic excitability in Almog and Hay model when a faster Ih inactivation was assumed. The experiments of S7 Fig were repeated using 4 times smaller time constants of Ih inactivation (τ∞) than in the original Hay and Almog models. See S7 Fig for details. E: Threshold current amplitudes for 2-ms square-pulse inputs at the apical dendrite at different distances from the soma using 4 times smaller time constants of Ih inactivation. Black: Hay-model control neuron, blue: Hay-model neuron with Ih blockage. The amplitudes are very similar to those with the original time constants (Fig 2G). (PDF) [file pcbi.1010506.s008.pdf]

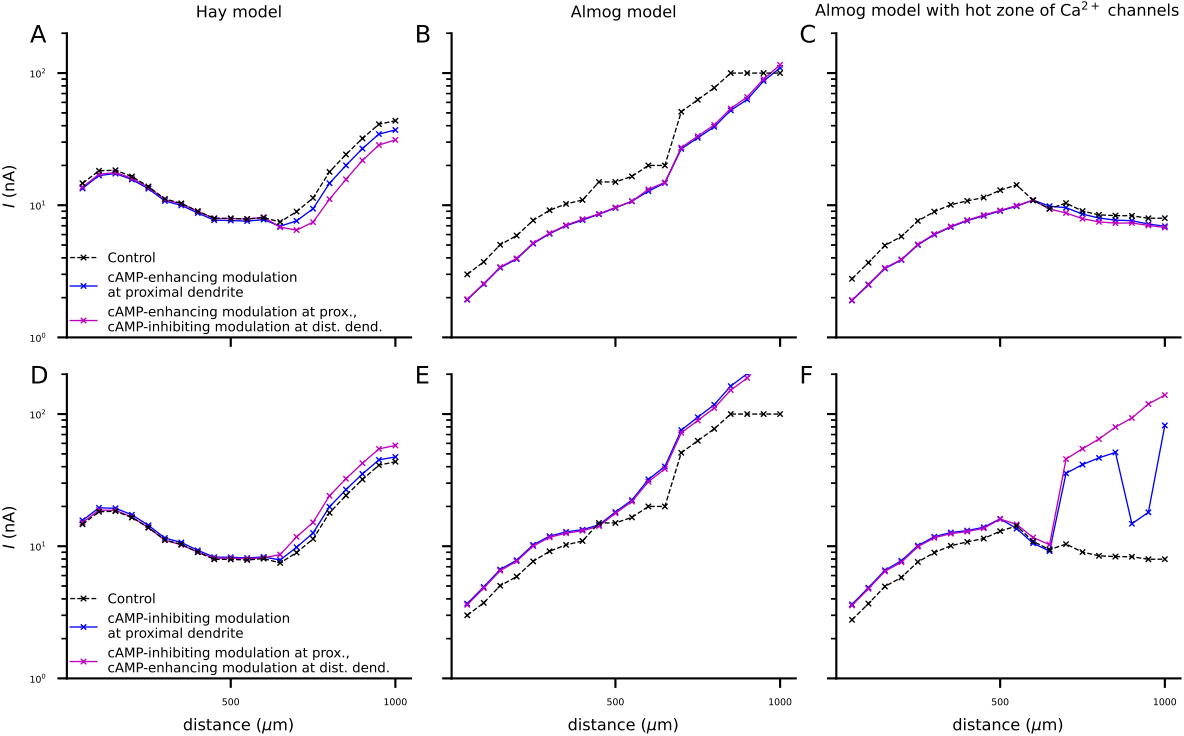

Supplement: S10 Fig — A–C: Threshold amplitude for a 2-ms current input applied to the apical dendrite at a given distance (x-axis) from soma according to Hay model (A), Almog model (B), or Almog model with a hot zone of LVA Ca2+ channels (C). Black: control neuron. Blue: neuron with proximal apical dendrite under cAMP-enhancing neuromodulation. Magenta: neuron with proximal apical dendrite under cAMP-enhancing neuromodulation and distal apical dendrite under cAMP-inhibiting neuromodulation. D–F: The experiment of (A)–(C) repeated with opposite modulation, i.e., control neuron (black) and neuron with cAMP-inhibiting modulation of proximal apical dendrite with (magenta) or without (blue) cAMP-enhancing modulation of the distal apical dendrite. (PDF) [file pcbi.1010506.s010.pdf]

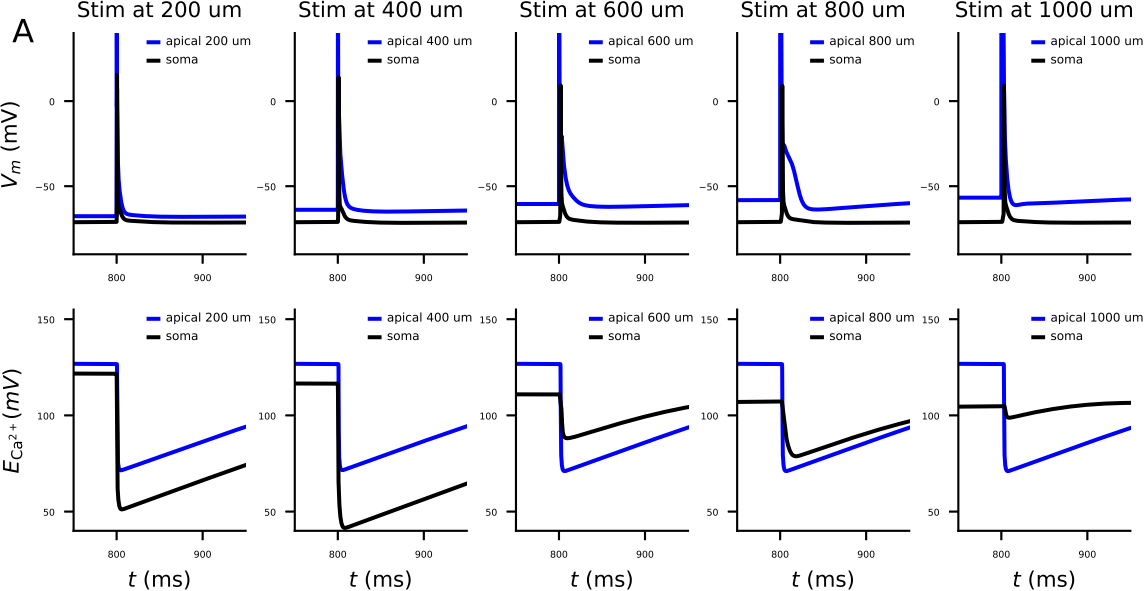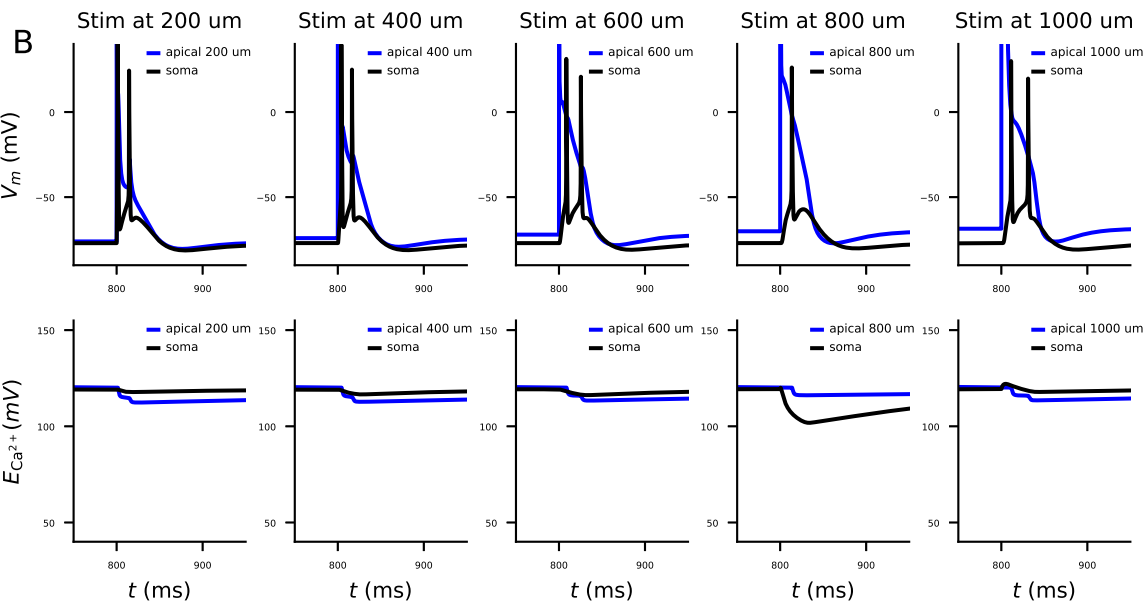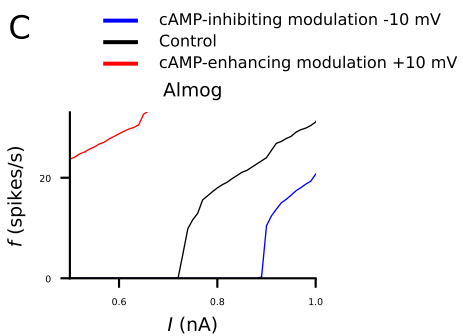

Supplement: S12 Fig — A: Membrane potential (upper panels) and Ca2+-channel reversal potential (lower panels) time series in response to short (0.2 ms) supra-threshold current stimuli at the apical dendrite at distances 200 (left)—1000 (right) μ according to the Almog model. The stimulus amplitude was 30 nA, except at the distance of 800 μ and 1000 μ amplitudes 100 and 300 nA, respectively, were used. B: Membrane potential (upper panels) and Ca2+-channel reversal potential (lower panels) time series in response to short (0.2 ms) supra-threshold current stimuli at the apical dendrite at distances 200 (left)—1000 (right) μ according to the Hay model. The stimulus amplitude was 30 nA, except at the distance of 1000 μ amplitude 100 nA was used. C: The experiments of Fig 1G were repeated using neuromodulatory voltage shifts of ±10 mV. (PDF) [file pcbi.1010506.s012.pdf]
