# Supplementary material for: Excitatory and inhibitory effects of HCN channel modulation on excitability of layer V pyramidal cells
Source: PLoS Comput Biol. 2022 Sep 13;18(9):e1010506. doi: 10.1371/journal.pcbi.1010506 (PMC9506642; doi:10.1371/journal.pcbi.1010506)
Supplement: S6 Fig — A: Predictions of the Almog model with a hot zone of Ca2+ channels. The upper left grid shows the threshold conductances for a set of 2000 excitatory synapses to induce an AP, and the lower right grid shows the factor by which the threshold conductance of the Ih-blocked neuron is larger (red) or smaller (blue) than that of the control neuron. See Fig 4F for details. B–C: Predictions of the Hay model for the cAMP-enhancing (B) or cAMP-inhibiting (C) neuromodulation compared to the non-modulated neuron. Upper left grid: The threshold conductances for a set of 2000 excitatory synapses to induce an AP in the Hay model. In each grid slot, the color of the upper right triangle indicates the threshold conductance in the control neuron whereas that of the lower left triangle indicates the threshold conductance in the neuron under cAMP-enhancing (B) or cAMP-inhibiting (C) neuromodulation. Lower right grid: The factor by which the threshold conductance of the neuron under cAMP-enhancing (B) or cAMP-inhibiting (C) neuromodulation is larger (red) or smaller (blue) than that of the control neuron. (PDF) [file pcbi.1010506.s006.pdf]

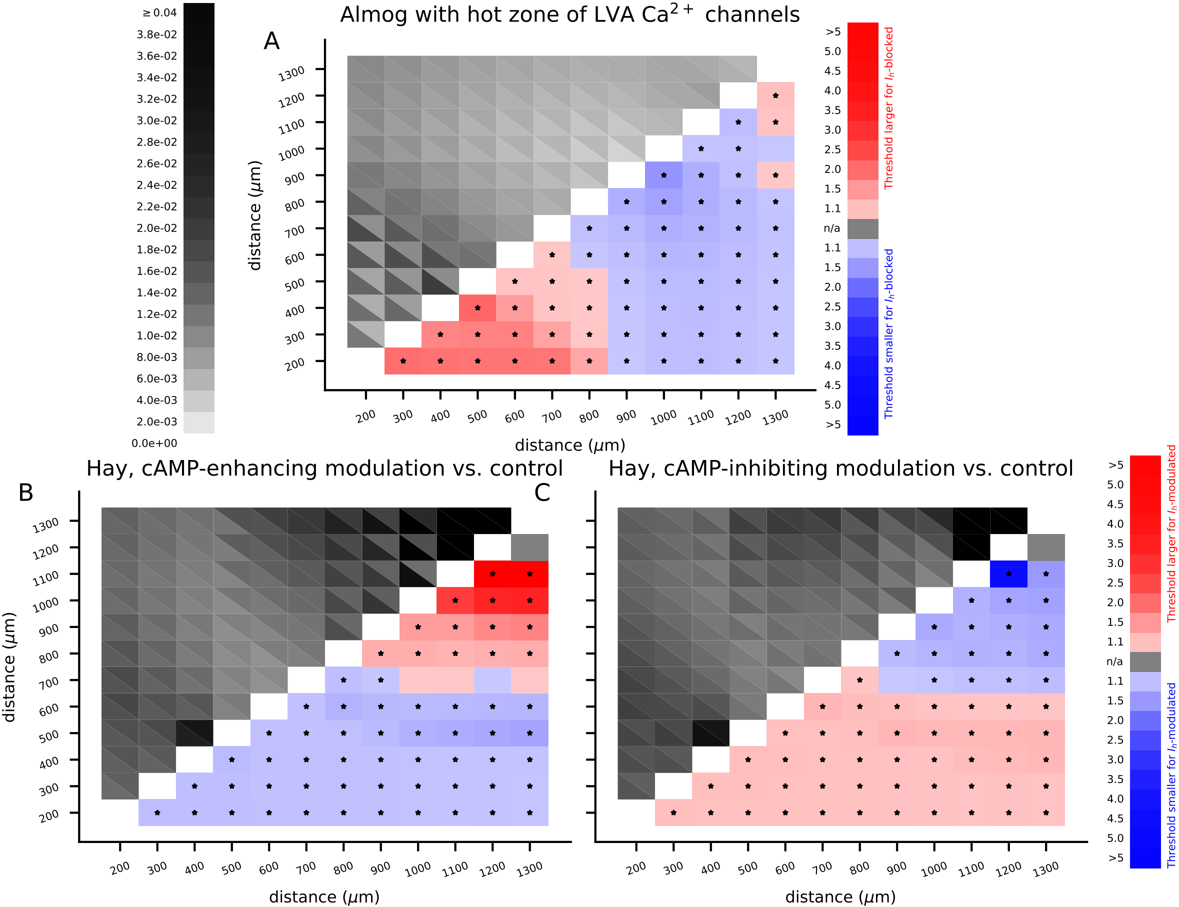

Almog with hot zone of LVA Ca^{2+} channels
A
B
C
Hay, cAMP-enhancing modulation vs. control
Hay, cAMP-inhibiting modulation vs. control
distance (μm)
Threshold larger for I_h-blocked
Threshold smaller for I_h-blocked
Threshold larger for I_h-modulated
Threshold smaller for I_h-modulated
≥ 0.04
0.0e+00
n/a
