# Supplementary material for: Excitatory and inhibitory effects of HCN channel modulation on excitability of layer V pyramidal cells
Source: PLoS Comput Biol. 2022 Sep 13;18(9):e1010506. doi: 10.1371/journal.pcbi.1010506 (PMC9506642; doi:10.1371/journal.pcbi.1010506)
Supplement: S7 Fig — A–B: Predictions of the Almog model with a hot zone of Ca2+ channels for the threshold currents in control neuron and neuron with Ih channels weakly modulated by cAMP-enhancing (+2 mV; A) or cAMP-inhibiting (-2 mV; B) neuromodulation. C–D: Predictions of the Hay model for the threshold currents in control neuron and neuron with Ih channels weakly modulated by cAMP-enhancing (+4 mV; C) or cAMP-inhibiting (-4 mV; D) neuromodulation. See Fig 4F for details. (PDF) [file pcbi.1010506.s007.pdf]

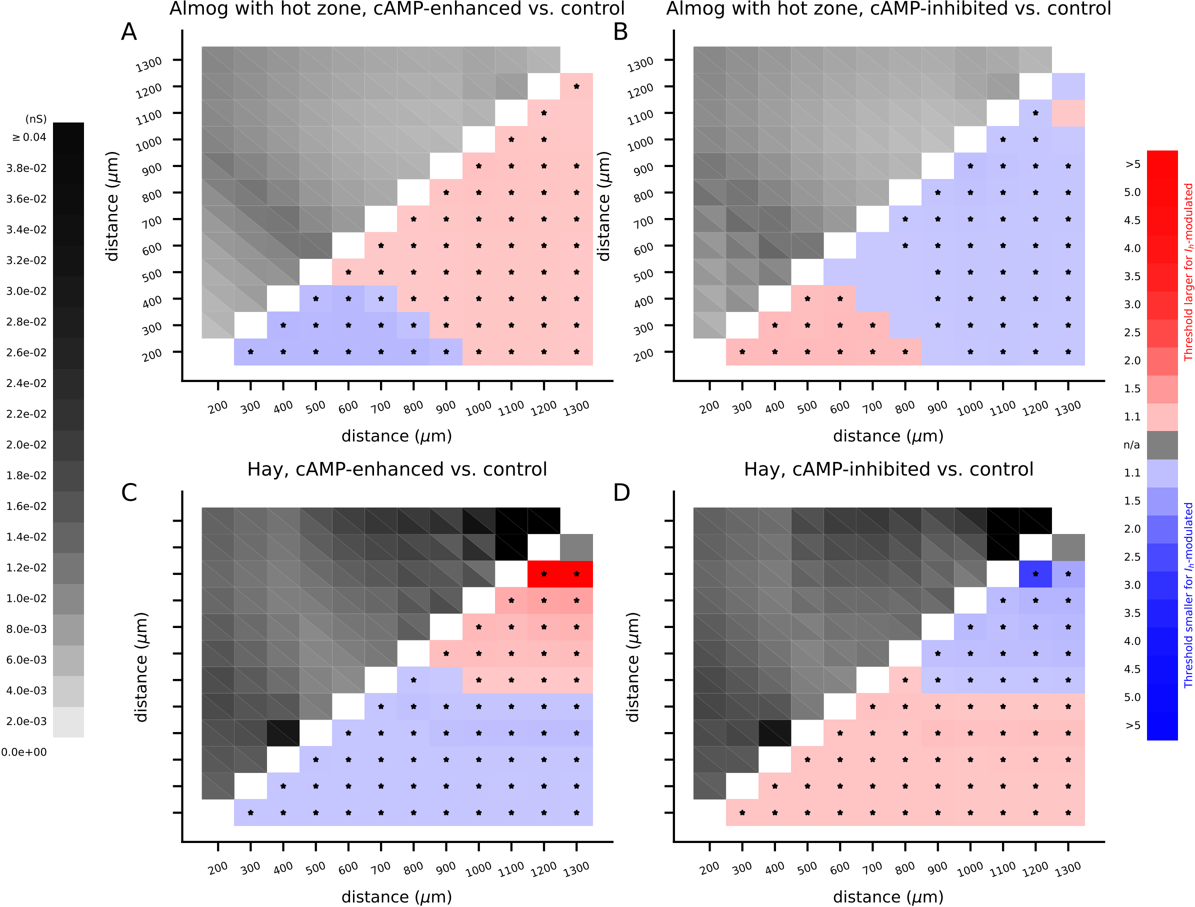
Almog with hot zone, cAMP-enhanced vs. control
Almog with hot zone, cAMP-inhibited vs. control
Hay, cAMP-enhanced vs. control
Hay, cAMP-inhibited vs. control
A
B
C
D
distance (μm)
(nS)
≥ 0.04
0.0e+00
n/a
Threshold larger for I_h-modulated
Threshold smaller for I_h-modulated
