# Supplementary material for: Excitatory and inhibitory effects of HCN channel modulation on excitability of layer V pyramidal cells
Source: PLoS Comput Biol. 2022 Sep 13;18(9):e1010506. doi: 10.1371/journal.pcbi.1010506 (PMC9506642; doi:10.1371/journal.pcbi.1010506)
Supplement: S11 Fig — The experiment of Fig 6 was repeated such that the one-sided neuromodulation (proximal dendrite modulated and distal dendrite unmodulated, i.e., the blue data of Fig 6) was replaced by the alternative one-sided neuromodulation (distal dendrite modulated, proximal dendrite unmodulated, green data). See Fig 6 for details. (PDF) [file pcbi.1010506.s011.pdf]

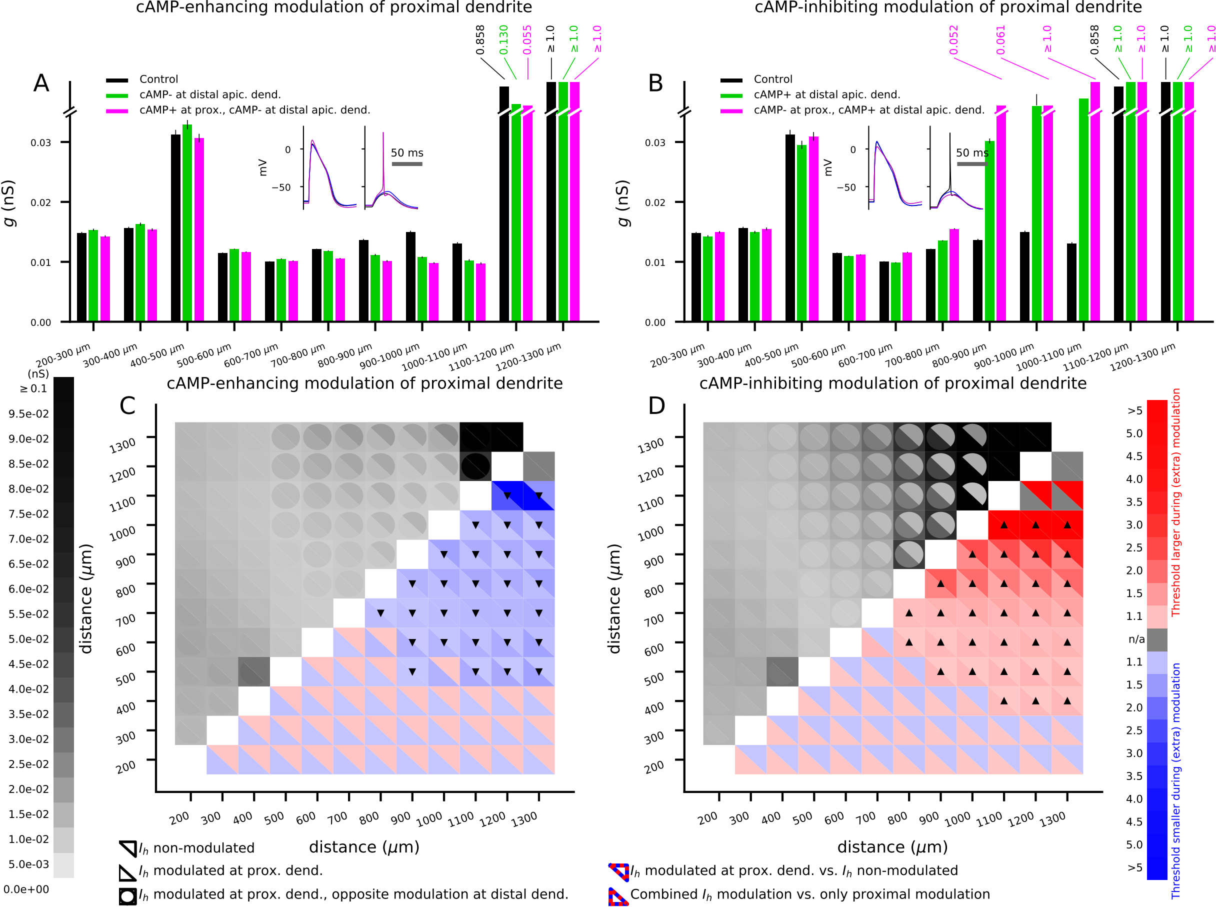

cAMP-enhancing modulation of proximal dendrite
cAMP-inhibiting modulation of proximal dendrite
A
Control
cAMP- at distal apic. dend.
cAMP+ at prox., cAMP- at distal apic. dend.
0.858
0.130
0.055
≥ 1.0
≥ 1.0
≥ 1.0
B
Control
cAMP+ at distal apic. dend.
cAMP- at prox., cAMP+ at distal apic. dend.
0.052
0.061
≥ 1.0
0.858
≥ 1.0
≥ 1.0
≥ 1.0
≥ 1.0
≥ 1.0
g (nS)
0.00
0.01
0.02
0.03
mV
0
−50
50 ms
200-300 μm
300-400 μm
400-500 μm
500-600 μm
600-700 μm
700-800 μm
800-900 μm
900-1000 μm
1000-1100 μm
1100-1200 μm
1200-1300 μm
(nS)
≥ 0.1
9.5e-02
9.0e-02
8.5e-02
8.0e-02
7.5e-02
7.0e-02
6.5e-02
6.0e-02
5.5e-02
5.0e-02
4.5e-02
4.0e-02
3.5e-02
3.0e-02
2.5e-02
2.0e-02
1.5e-02
1.0e-02
5.0e-03
0.0e+00
C
D
distance (μm)
200
300
400
500
600
700
800
900
1000
1100
1200
1300
>5
5.0
4.5
4.0
3.5
3.0
2.5
2.0
1.5
1.1
n/a
1.1
1.5
2.0
2.5
3.0
3.5
4.0
4.5
5.0
>5
Threshold larger during (extra) modulation
Threshold smaller during (extra) modulation
I_h non-modulated
I_h modulated at prox. dend.
I_h modulated at prox. dend., opposite modulation at distal dend.
I_h modulated at prox. dend. vs. I_h non-modulated
Combined I_h modulation vs. only proximal modulation
