# Supplementary material for: Excitatory and inhibitory effects of HCN channel modulation on excitability of layer V pyramidal cells
Source: PLoS Comput Biol. 2022 Sep 13;18(9):e1010506. doi: 10.1371/journal.pcbi.1010506 (PMC9506642; doi:10.1371/journal.pcbi.1010506)
Supplement: S9 Fig — A–D: The four panels show the Hay-model predictions for the effects of cAMP-enhancing (A,C) or cAMP-inhibiting (B,D) neuromodulation on the AP thresholds of apical dendritic stimulation in the presence of simultaneous glutamatergic (A–B) or GABAergic (C–D) stimulation of the basal dendrite. Upper left grids: The threshold conductances for a set of 2000 excitatory synapses to induce an AP in the Hay model. In each grid slot, the color of the upper right triangle indicates the threshold conductance in the control neuron whereas that of the lower left triangle indicates the threshold conductance in the neuron under cAMP-enhancing (A–B) or cAMP-inhibiting (C–D) neuromodulation. Lower right grids: The factor by which the threshold conductance of the neuron under cAMP-enhancing (A,C) or cAMP-inhibiting (B,D) neuromodulation is larger (red) or smaller (blue) than that of the control neuron. (PDF) [file pcbi.1010506.s009.pdf]

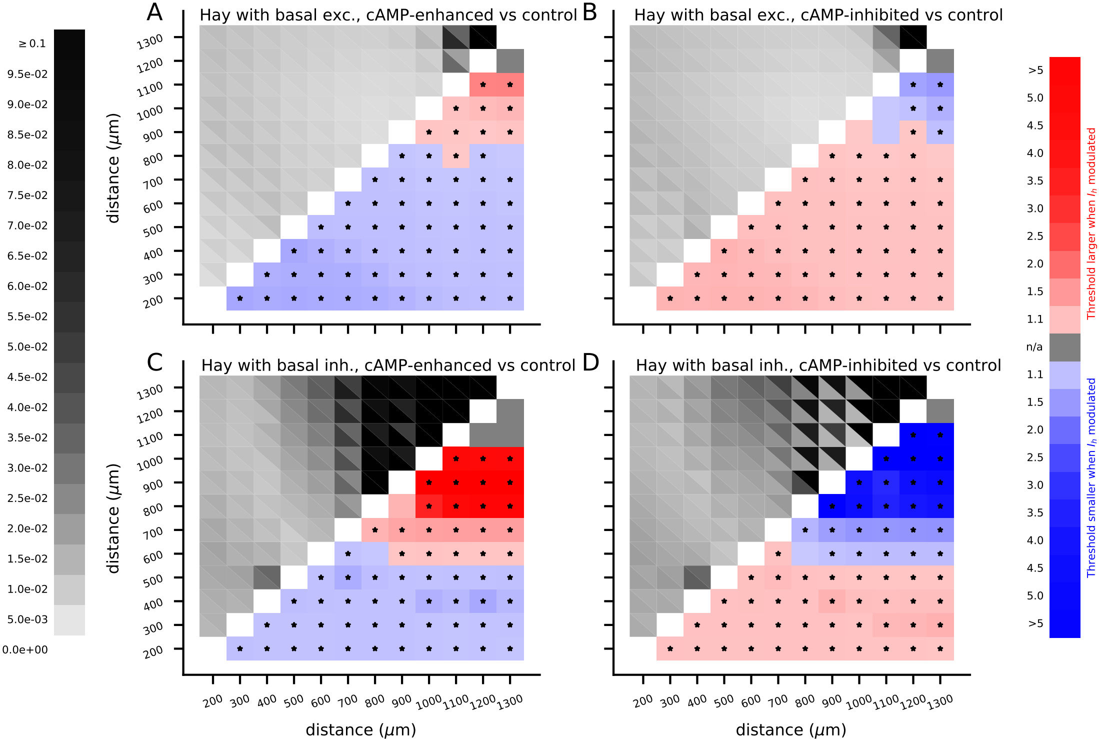

A
Hay with basal exc., cAMP-enhanced vs control
B
Hay with basal exc., cAMP-inhibited vs control
C
Hay with basal inh., cAMP-enhanced vs control
D
Hay with basal inh., cAMP-inhibited vs control
distance (μm)
distance (μm)
200 300 400 500 600 700 800 900 1000 1100 1200 1300
≥ 0.1
9.5e-02
9.0e-02
8.5e-02
8.0e-02
7.5e-02
7.0e-02
6.5e-02
6.0e-02
5.5e-02
5.0e-02
4.5e-02
4.0e-02
3.5e-02
3.0e-02
2.5e-02
2.0e-02
1.5e-02
1.0e-02
5.0e-03
0.0e+00
>5
5.0
4.5
4.0
3.5
3.0
2.5
2.0
1.5
1.1
n/a
1.1
1.5
2.0
2.5
3.0
3.5
4.0
4.5
5.0
>5
Threshold larger when I_h modulated
Threshold smaller when I_h modulated
