# Supplementary material for: Excitatory and inhibitory effects of HCN channel modulation on excitability of layer V pyramidal cells
Source: PLoS Comput Biol. 2022 Sep 13;18(9):e1010506. doi: 10.1371/journal.pcbi.1010506 (PMC9506642; doi:10.1371/journal.pcbi.1010506)
Supplement: Attachment — Submitted filename: Maki_Marttunen2022_HCN_simulation_Response.pdf [file pcbi.1010506.s014.pdf]

(1) Models of HCN channel and the effects of neuromodulators

Reviewer #1 comment:

The authors refer to two experimental studies on which their implementation of voltage dependency of HCN channel conductance critically depends (Zhao et al., 2016 and Byczkowicz et al., 2019). These studies examined the impacts of acetylcholine (ACh) and dopamine on the striatal neuron and cerebellar mossy fiber boutons, both of which primarily expressing HCN2 channels.
However, the authors of this study examined the behavior of the cortical neuron. There is co-expression of HCN1 and HCN2 channels in pyramidal cells. The discrepancy between relevant HCN subtypes raises a major concern.
HCN1 channels and HCN2 channels have significantly different activation speeds and sensitivities to cyclic AMP (He et al., 2014, PMID: 24184323). When co-expressed in the same cell, these properties differ depending on the fraction of subtypes (Chen et al., 2001, PMID: 11331358). Therefore, the degree of modulation used in this study's computational model is not realistic and potentially far greater than in vivo conditions. This has critical implications for the veracity of their computational model because one of their major findings depends on the shifted voltage dependency.
The authors must clarify the fraction of HCN1 and HCN2 channels in L5PC and accordingly control the HCN channel properties in their model. If they find similar results as to what was reported here, then it's interesting.

Authors response:

We thank the reviewer for his/her comment. We agree that it is important to discuss the subunit-dependence of the HCN channel modulation. The cAMP-dependence of HCN1-type channels is indeed weaker than that of HCN2-type, mid-activation voltage being shifted by cAMP approximately by +4 mV in the former and +17 mV in the latter. However, the findings of Chen et al. (J Gen Physiol. 2001 May 1; 117(5): 491–504) reveal that heteromultimeric HCN1-HCN2 channels are also strongly modulated (mid-activation voltage shifted by +14 mV) by cAMP. This is a stronger effect than the shifts used in our work (+5 or +10 mV). It is very likely that L5PCs, which are known to express both HCN1 and HCN2 genes, also express the heteromultimeric channels (see e.g. Ulens and Tytgat 2001, PMID: 11133998) but to our knowledge, this has not yet been fully demonstrated. In other words, it is difficult to estimate the HCN1:HCN2 ratios in pyramidal cells and even more difficult the ratio of HCN1 homomers:HCN2 homomers:HCN1-HCN2 heteromers. We therefore opted to repeat our simulations by applying a smaller (±2 or ±4 mV) shift which would correspond to HCN1-only L5PCs. We show that our conclusions hold even in the unlikely case that HCN1-HCN2 heteromers do not exist in the pyramidal cells. By contrast, the case of HCN2-dominant L5PCs would be accounted for by our original results for ±10 mV shifts or even more radical modulations.

Reviewer #1 response:

I initially expressed concerns about the difference in the HCN1/HCN2 ratio between the model and the physiological pyramidal neurons. This discrepancy has the potential to greatly affect the results because these two subtypes show significantly different activation speeds and voltage dependencies. I therefore

recommended that the authors compare their HCN1/HCN2 ratio to that which is observed experimentally and then systematically control the HCN channel properties in their model.

The authors appropriately and convincingly addressed the concern of different cAMP sensitivities between HCN1 and HCN2. Because no experimental study has empirically evaluated the in vivo ratio of HCN1/HCN2 in L5PCs, the authors addressed this concern by testing an extreme condition of strictly HCN1 channels in their model. The authors added a new result with minor voltage dependency shift reflecting the lower cAMP sensitivity of HCN1. The authors also address the possibility for heteromultimeric channels, the presence of which would not be expected to change the crux of their results.

However, I also noted that HCN1 and HCN2 differ not only in their sensitivity to cAMP, but also their activation speed (see figure 1B in Chen et al., 2001, and figure 1B in Ulens and Tytgat, 2001, both papers are already in the reference list). HCN1 channels activate several times faster than HCN2 channels. This difference potentially affects the spike generation in the short time window that is tested in many simulations of this study. Therefore, the authors need to adjust the channel kinetics for heteromeric HCN channels at a minimum (or show that all results are consistent with the results in HCN1 only condition, which represents a greater extreme) in addition to the cAMP sensitivity.

(2) Concentrations of ACh and dopamine

Reviewer #1 comment:

In the referenced studies, the modulations of voltage dependency were performed by either controlling intracellular cAMP through a glass pipette during the patch-clamp recording or by the perfusion of the antagonist in the extracellular solution. The concentration of neuromodulators reported by the submitting authors is likely beyond the physiological operating range of cortical neurons reported previously.
For example, Hironaka et al. (2001) reported that the ACh concentration in the rat prefrontal cortex ranged from 10 nM to 30 nM (PMID: 11368961). On the other hand, the dose of ACh used in the referenced work (Zhao et al., 2016) used 50 µM of ACh. Additionally, Spühler and Hauri (2013) developed a simulation allowing in silico estimation of dopamine concentrations in prefrontal cortex (PMID: 23951205). Spühler and Hauri found that the concentration of dopamine in the prefrontal cortex likely falls between 9.5 nM to 250 nM in the steady state and 23.4 nM to 989 nM with 26 Hz phasic dopamine release. The submitting authors instead refer to Byczkowicz et al. (2019), which used 200 µM of dopamine in their study.
Zhao et al. (2016) and Byczkowicz et al. (2019) used doses of Ach and Dopamine, respectively, that are more than 200 times high than what was previously reported as possible in vivo. These doses may represent a non-physiological perturbation that are not ideal for a simulation that attempts to reconstruct normal, physiological spiking activity.
The submitting authors need to adjust the effects of neuromodulators based on physiological concentrations. If they find similar results as to what was reported here, then it’s interesting.

Authors response 2-1:

We thank the reviewer for this comment. There are indeed at least two ways of experimentally studying the effects of cAMP-coupled neuromodulators on Ih activation: either applying the neuromodulator (or corresponding agonist) to the extracellular medium, or directly applying cAMP (or cAMP pathway inhibitor) into the intracellular medium. Apart from Zhao et al. 2016, all our references use the latter approach (although, as noted by the reviewer, Byczkowicz et al. also applied dopamine in a related experiment; and Zhao et al. also find similar results using the latter approach) but similar magnitudes for voltage shifts have been reported using the former approach. For example., Wu and Hablitz 2005 (PMC6725283) reported a depolarizing shift of the half-inactivation voltage of 7 mV by application of 30 uM dopamine, while our references found shifts ranging from 4 to 17 mV by increased intracellular cAMP.

Reviewer #1 response 2-1:

I originally pointed out the discrepancy in the neurotransmitter dose between physiological and experimental conditions (and the potential gap in the electrophysiological effect between them). Note that the experimental conditions are not necessarily physiological conditions. Experiments performed in vitro (i.e. patch clamp recordings in slice) do not represent a physiological condition that can be studied in vivo (i.e. microdialysis in live animal cortex). For example, Hironaka et al. (2001) recorded ***in vivo*** Ach concentrations, while Zhao et al. (2016) examined HCN channel properties ***in vitro*** using a far larger

dose of Ach in the extracellular solution than ever observed in a live animal. Therefore, I noted that it was not appropriate for the authors to use a similar degree of voltage shift reported by Zhao et al. 2016.

In the authors' response, the authors referenced the similar magnitudes of voltage shifts between two experimental conditions (extracellular application of agonist and intracellular perfusion or inside-out patch clamp). However, they did not show if the dose of neurotransmitters in such studies are physiological. Therefore, the authors did not address my concern. If the authors intend to simulate the physiological condition, parameters must be within the physiologically realistic range.

Authors response 2-2:

As for the neuromodulator concentrations used in our references, it is true that ACh concentrations measured in the extracellular medium in vivo are within the nanomolar range (as shown in e.g. Hironaka et al. 2001 mentioned by the reviewer), which is much smaller than the in vitro experiment of Zhao et al., 2016. However, one should keep in mind that, in vivo, the local, transient concentrations of ACh are much higher in the synaptic cleft and other confined volumes near the post-synaptic neuron's dendrites than the measured global concentrations in the extracellular medium. In Barberis et al. 2011 (PMC3123770), it was estimated that an ejection of 4000 GABA molecules implies a concentration pulse of hundreds of μM in the synaptic cleft. Similar estimates were made for ACh in Aidoo, A. Y., & Ward, K. (2006), Mathematical and computer modelling, 44(9-10), 952-962. By contrast, a neurotransmitter concentration of a nanomolar magnitude in the synaptic cleft would correspond to a handful of neurotransmitter molecules. Thus, we do not consider the bath concentrations used in our references exaggeratingly large (especially since the experiments that directly altered intracellular cAMP concentrations yielded similar ranges of voltage shifts), although we agree with the reviewer that further research with varied concentrations would be enlightening.

Reviewer #1 response 2-2:

I expressed the concern that the authors' simulations used a degree of voltage shift obtained from experiments in which the dose of neurotransmitter was more than 200 times higher than the physiological dose.

To address this concern, the authors explained that the dose of neurotransmitters inside the synaptic cleft is higher than in the extracellular space. This idea is reasonable if the neurotransmitter is released and the receptor for the same neurotransmitter exists in the same synapse. However, this model is often not applicable to modulatory neurotransmitters and their receptors. The authors should refer to work on the synapses and axon terminals of the pyramidal neurons as addressed by electron microscopy (see below).

In the case of M2-type muscarinic receptor, Mrzljak et al. reported that the M2 receptors localize exclusively at asymmetric synapses in pyramidal neurons (Mrzljak et al., 1998, PMID: 9456180). However, cholinergic synapses in pyramidal neurons are not asymmetric synapses (Mrzljak et al., 1995, PMID: 7673486). Therefore, most M2 receptors do not receive the acetylcholine released in the same synapse. These observations indicate that cholinergic neurotransmission to M2 receptors in pyramidal

neurons occurs via volume neurotransmission. Dopamine is released in a similar manner, and the D1 receptor is also not localized to the dopaminergic terminal (Smiley et al., 1994, PMID: 7911245).

Therefore, we cannot expect a high dose of acetylcholine and dopamine in the M2 receptor-positive and D1 receptor-positive synaptic clefts in the cortical pyramidal neuron. The authors need to use the extra-synaptic dose for both acetylcholine and dopamine and adjust the degree of voltage shift in the model accordingly. In my first comment I linked papers that directly measure the extracellular concentrations for acetylcholine and a theoretical model estimating dopamine concentrations. I expect the authors to repeat their simulations using these estimates, which are, to my knowledge, our best guess of the physiological operating range of these two neuromodulators. If the authors know of another reference for in vivo extracellular (i.e. physiological) concentrations then I could be convinced of a higher dose.

Authors response 2-3:

To show the relevance of the chosen range of voltage shifts, we have included references to additional experiments supporting our use of 5-10 mV shifts, namely Chen et al. 2001 (mentioned by the reviewer), Ulens and Tytgat 2001, Ludwig et al. 1998, Wang et al. 2001, Ko et al. 2016 – see our response above. To further highlight the fact that smaller neuromodulator concentrations can lead to large effects, we also refer to a study where 10 uM serotonin application had significant effects on L5PC excitability, mediated by Ih current.

Reviewer #1 response 2-3:

The authors added a description of the serotonergic modulation of the HCN1 channel.

For the serotonergic transmission, Séguéla et al. found that only 38% of serotonergic varicosities formed a synaptic structure on dendrites and spines in the multiple cerebral cortices, and the remaining varicosities are nonjunctional (Séguéla et al., 1989, PMID: 2808757). Later, Smiley and Goldman-Rakic reconstructed the serotonergic synapses in the prefrontal cortex and reported that 92% of serotonergic synapses were made on the GABAergic interneuron and only 8% of synapses were on the dendritic shafts that showed features of pyramidal neurons (Smiley and Goldman-Rakic, 1996, PMID: 8698902). Therefore, pyramidal neurons have some serotonergic terminals on the dendritic shaft, although they are not major targets of serotonergic projection. However, DeFelipe et al. showed the intense exclusive labeling of 5-HT 1a receptors at the soma-axon compartment of the pyramidal neuron, and, in most labeled neurons, only the axons were labeled (DeFelipe et al., 2001, PMID: 11283956).

This discrepancy between the location of serotonergic synapses found on the dendritic shaft and the location of the receptor at the soma-axon shows that serotonergic transmission to pyramidal neurons is in a **paracrine** manner. As such, we would not expect to see a high concentration of serotonin gathered in a synaptic cleft. Therefore, without experimental evidence or a convincing computational study, 10 μM of serotonin in the external solution should not be considered a physiological condition.
