# Supplementary material for: Excitatory and inhibitory effects of HCN channel modulation on excitability of layer V pyramidal cells
Source: PLoS Comput Biol. 2022 Sep 13;18(9):e1010506. doi: 10.1371/journal.pcbi.1010506 (PMC9506642; doi:10.1371/journal.pcbi.1010506)
Supplement: Attachment — Submitted filename: reply_to_reviewers.docx [file pcbi.1010506.s013.docx]

Dear Editors,

We are resubmitting our manuscript “Excitatory and inhibitory effects of HCN channel modulation on excitability of layer V pyramidal cells”. We have made substantial changes to the content of the manuscript to address the concerns of the reviewers, and we believe the manuscript is significantly improved in consequence. Namely, we have 1) redesigned our figures to increase their readability and attract further analysis by readers, 2) presented support for our conclusions regardless of the exact HCN1 vs HCN2 proportion in layer V pyramidal cells, and 3) extended the discussion on interplay between I_h_ and LVA Ca^2+^ channels in layer V pyramidal cells (new Section 4.2). Please see below for our point-by-point addressing of the reviewers’ comments (reviewers’ comments in red, our reply in black, and quotations of the revised manuscript in blue).

On behalf of the authors,

Tuomo Mäki-Marttunen

Reviewer #1: 
1. Models of HCN channel and the effects of neuromodulators
The authors refer to two experimental studies on which their implementation of voltage dependency of HCN channel conductance critically depends (Zhao et al., 2016 and Byczkowicz et al., 2019). These studies examined the impacts of acetylcholine (ACh) and dopamine on the striatal neuron and cerebellar mossy fiber boutons, both of which primarily expressing HCN2 channels.
However, the authors of this study examined the behavior of the cortical neuron. There is co-expression of HCN1 and HCN2 channels in pyramidal cells. The discrepancy between relevant HCN subtypes raises a major concern.
HCN1 channels and HCN2 channels have significantly different activation speeds and sensitivities to cyclic AMP (He et al., 2014, PMID: 24184323). When co-expressed in the same cell, these properties differ depending on the fraction of subtypes (Chen et al., 2001, PMID: 11331358). Therefore, the degree of modulation used in this study’s computational model is not realistic and potentially far greater than in vivo conditions. This has critical implications for the veracity of their computational model because one of their major findings depends on the shifted voltage dependency.
The authors must clarify the fraction of HCN1 and HCN2 channels in L5PC and accordingly control the HCN channel properties in their model. If they find similar results as to what was reported here, then it’s interesting.

We thank the reviewer for his/her comment. We agree that it is important to discuss the subunit-dependence of the HCN channel modulation. The cAMP-dependence of HCN1-type channels is indeed weaker than that of HCN2-type, mid-activation voltage being shifted by cAMP approximately by +4 mV in the former and +17 mV in the latter. However, the findings of Chen et al. (J Gen Physiol. 2001 May 1; 117(5): 491–504) reveal that heteromultimeric HCN1-HCN2 channels are also strongly modulated (mid-activation voltage shifted by +14 mV) by cAMP. This is a stronger effect than the shifts used in our work (+5 or +10 mV). It is very likely that L5PCs, which are known to express both HCN1 and HCN2 genes, also express the heteromultimeric channels (see e.g. Ulens and Tytgat 2001, PMID: 11133998) but to our knowledge, this has not yet been fully demonstrated. In other words, it is difficult to estimate the HCN1:HCN2 ratios in pyramidal cells and even more difficult the ratio of HCN1 homomers:HCN2 homomers:HCN1-HCN2 heteromers. We therefore opted to repeat our simulations by applying a smaller (±2 or ±4 mV) shift which would correspond to HCN1-only L5PCs. We show that our conclusions hold even in the unlikely case that HCN1-HCN2 heteromers do not exist in the pyramidal cells. By contrast, the case of HCN2-dominant L5PCs would be accounted for by our original results for ±10 mV shifts or even more radical modulations.

We have now discussed this issue in Methods, Results and Discussion., On p. 5 (Methods, Section 2.3) we write:

To model weakly cAMP-dependent Ih channels (HCN1 homomeric channels) in Fig. S7, we used smaller half-inactivation voltage shifts, namely ±4 mV and ±2 mV in Hay and Almog models, respectively.

On p. 11 (Results) we write:

The statistical significance of these results remained even if we assumed a weaker, HCN1-like modulation (Fig. S7).

On p. 18 (Discussion) we write:

The main body of our results was obtained assuming a substantial voltage shift typical to heterotetrameric HCN1/HCN2 channels (half-inactivation voltage shifted by 7–14 mV by increased cAMP [Chen et al., 2001, Ulens and Tytgat, 2001]) and homomeric HCN2 channels (half-inactivation voltage shifted by 12–17 mV by increased cAMP [Ludwig et al., 1998, Wang et al., 2001, Byczkowicz et al., 2019]) — we modelled the effects of dopaminergic modulation using a half-inactivation voltage shift of 5–10 mV. However, for homomeric HCN1 channels shifts of 2 mV [Santoro et al., 1998] and 4.3 mV [Wang et al., 2001] have been observed.

Later, on the same page, we write:

Both HCN1 and HCN2 are excpressed in L5PCs [Lörincz et al., 2002, Christophe et al., 2005], and it is likely – although not explicitly shown – that they are co-localized and form heteromeric Ih channels. Our analysis carried out with a weaker cAMP sensitivity (half-inactivation voltage shifted by ±2–4 mV) confirms that our results on the dopaminergic and cholinergic modulation of L5PC excitability hold, although with smaller amplitudes, even if all Ih channels were of the HCN1-type (Figure S7). In sum, although I_h_ channel subunit composition affects the magnitude of the effect of neuromodulation, the directions of the effects of neuromodulation, as summarized in Table 2, are not affected by the type of I_h_ channel.

2. Concentrations of ACh and dopamine
In the referenced studies, the modulations of voltage dependency were performed by either controlling intracellular cAMP through a glass pipette during the patch-clamp recording or by the perfusion of the antagonist in the extracellular solution. The concentration of neuromodulators reported by the submitting authors is likely beyond the physiological operating range of cortical neurons reported previously.
For example, Hironaka et al. (2001) reported that the ACh concentration in the rat prefrontal cortex ranged from 10 nM to 30 nM (PMID: 11368961). On the other hand, the dose of ACh used in the referenced work (Zhao et al., 2016) used 50 μM of ACh. Additionally, Spühler and Hauri (2013) developed a simulation allowing in silico estimation of dopamine concentrations in prefrontal cortex (PMID: 23951205). Spühler and Hauri found that the concentration of dopamine in the prefrontal cortex likely falls between 9.5 nM to 250 nM in the steady state and 23.4 nM to 989 nM with 26 Hz phasic dopamine release. The submitting authors instead refer to Byczkowicz et al. (2019), which used 200 μM of dopamine in their study.
Zhao et al. (2016) and Byczkowicz et al. (2019) used doses of Ach and Dopamine, respectively, that are more than 200 times high than what was previously reported as possible in vivo. These doses may represent a non-physiological perturbation that are not ideal for a simulation that attempts to reconstruct normal, physiological spiking activity.
The submitting authors need to adjust the effects of neuromodulators based on physiological concentrations. If they find similar results as to what was reported here, then it’s interesting.

We thank the reviewer for this comment. There are indeed at least two ways of experimentally studying the effects of cAMP-coupled neuromodulators on I_h_ activation: either applying the neuromodulator (or corresponding agonist) to the extracellular medium, or directly applying cAMP (or cAMP pathway inhibitor) into the intracellular medium. Apart from Zhao et al. 2016, all our references use the latter approach (although, as noted by the reviewer, Byczkowicz et al. also applied dopamine in a related experiment; and Zhao et al. also find similar results using the latter approach) but similar magnitudes for voltage shifts have been reported using the former approach. For example., Wu and Hablitz 2005 (PMC6725283) reported a depolarizing shift of the half-inactivation voltage of 7 mV by application of 30 uM dopamine, while our references found shifts ranging from 4 to 17 mV by increased intracellular cAMP. We now mention this on p. 18:

It should be noted that although the above data and the data on HCN2-channel modulation [Byczkowicz et al., 2019] were based on measured effects of direct increase of intracellular cAMP, voltage shifts of similar magnitude have been observed when dopamine was applied in the extracellular medium (e.g., 30 μM dopamine shifted the half-inactivation voltage of I_h_ currents in layer I interneurons by +7 mV in [Wu and Hablitz, 2005]).

As for the neuromodulator concentrations used in our references, it is true that ACh concentrations measured in the extracellular medium *in vivo* are within the nanomolar range (as shown in e.g. Hironaka et al. 2001 mentioned by the reviewer), which is much smaller than the *in vitro* experiment of Zhao et al., 2016. However, one should keep in mind that, *in vivo*, the local, transient concentrations of ACh are much higher in the synaptic cleft and other confined volumes near the post-synaptic neuron’s dendrites than the measured global concentrations in the extracellular medium. In Barberis et al. 2011 (PMC3123770), it was estimated that an ejection of 4000 GABA molecules implies a concentration pulse of hundreds of $\mu$M in the synaptic cleft. Similar estimates were made for ACh in Aidoo, A. Y., & Ward, K. (2006), *Mathematical and computer modelling*, *44*(9-10), 952-962. By contrast, a neurotransmitter concentration of a nanomolar magnitude in the synaptic cleft would correspond to a handful of neurotransmitter molecules. Thus, we do not consider the bath concentrations used in our references exaggeratingly large (especially since the experiments that directly altered intracellular cAMP concentrations yielded similar ranges of voltage shifts), although we agree with the reviewer that further research with varied concentrations would be enlightening.

To show the relevance of the chosen range of voltage shifts, we have included references to additional experiments supporting our use of 5-10 mV shifts, namely Chen et al. 2001 (mentioned by the reviewer), Ulens and Tytgat 2001, Ludwig et al. 1998, Wang et al. 2001, Ko et al. 2016 – see our response above. To further highlight the fact that smaller neuromodulator concentrations can lead to large effects, we also refer to a study where 10 uM serotonin application had significant effects on L5PC excitability, mediated by I_h_ current. In addition to the sentences added in response to the comment above, on p. 17 we write:

Moreover, similar to cholinergic M2 receptors, serotonergic 5HT1A receptors (Gi/o coupled) shift the half-inactivation voltage of Ih currents toward hyperpolarized potentials [Ko et al., 2016], and these receptors mediated an inhibitory net effect in a subgroup of prefrontal cortical L5PCs [Elliott et al., 2018].

To discuss the future directions for detailed modelling of I_h_ channels, we now write on p. 22:

Furthermore, modelling of intracellular cAMP concentration and its effects on the I_h_ activation would be beneficial since the concentration of cAMP varies across dendrites [Katz and Clemens, 2001], and thus I_h_ channels in one part of the dendritic tree may be more prone to negative cAMP modulation and others to positive one.

1. Authors may need to explain the model of voltage-dependent calcium channels in the methods section because they modulate the parameters of these channels in the results 3.2.

We have now added the description of these channels. On p. 3-4 we write:

The dynamics of the I current, gated by the inactivation variable h as $I_{h}=\bar{g_{h}}h\left( E_{h}-V_{m} \right)$, is described as follows. In both models, the variable *h* obeys the equation

$$\frac{dh}{dt}=\frac{1}{\tau_{\infty}}\left( h_{\infty}-h \right)$$

In the Hay model, $h_{\infty}$and $\tau_{\infty}$are described as

$$\alpha_{h}=-\frac{v_{\text{off},a}-v}{\tau_{a}}\frac{1}{exp\left( -\left( v_{\text{off},a}-V_{m} \right)/v_{\text{slo}} \right)-1}$$

$$\beta_{h}=\frac{exp\left( -\left( v_{\text{off},b}-V_{m} \right)/v_{\text{slo},b} \right)}{\tau_{b}}$$

$h_{\infty}=\frac{\alpha_{h}}{\alpha_{h}+\beta_{h}}$	(1)

$$\tau_{\infty}=\frac{1}{\alpha_{h}+\beta_{h}}$$

with $v_{\text{off},a}$ = -154.9 mV, $v_{\text{slo},a}$ = 11.9 mV, $\tau_{a}$ = 155.521 ms, $v_{\text{off},b}$ = 0.0 mV, $v_{\text{slo},b}$ = 33.1 mV, and $\tau_{b}$= 5.18135 ms. In the Almog model, $h_{\infty}$and $\tau_{\infty}$are described as

$h_{\infty}=\frac{1}{1+exp\left( \left( V_{m}-v_{\text{off}} \right)/v_{\text{slo}} \right)}$	(2)

$$\tau_{\infty}=\frac{1}{t_{adj}\left( \frac{1}{t_{0}}exp\left( \left( v_{\text{off},t1}-V_{m} \right)/v_{\text{slo},t1} \right)+\frac{1}{t_{1}}exp\left( -\left( v_{\text{off},t2}-V_{m} \right)/v_{\text{slo},t2} \right) \right)}$$

$$t_{adj}=q_{10}^{\left( T-{22}^{\circ}\text{C} \right)/{10}^{\circ}\text{C}}$$

where $v_{\text{off}}$ = -154.9 mV, $v_{\text{slo}}$ = 6 mV, $t_{0}$ = 2542.5883549 ms, $t_{1}$ = 11.40250855 ms, $v_{\text{off},t1}$ = 0 mV, $v_{\text{off},t2}$ = 0 mV, $v_{\text{slo},t1}$ = 40.1606426 mV, $v_{\text{slo},t2}$ = 16.1290323 mV, $q_{1}0$ = 2.3, and $T$ = 34$^{\circ}$C. The reversal potential of the *I_h_* current was more depolarized in the Almog model (*E_h_* = -33 mV) than in the Hay model (*E_h_* = -45 mV).

The dynamics of the LVA Ca^2+^ current in the Hay model, gated by activation and inactivation variables $m$

and $h$ as $I_{CaLVA}=\bar{g_{CaLVA}}m^{2}h\left( E_{Ca^{2+}}-V_{m} \right)$, is described as follows:

$\frac{dm}{dt}=\frac{1}{\tau_{m,\infty}}\left( m_{\infty}-m \right)$	(3)

$\frac{dh}{dt}=\frac{1}{\tau_{h,\infty}}\left( h_{\infty}-h \right)$	(4)

$m_{\infty}=\frac{1}{1+exp\left( \left( v_{\text{off},m}-V_{m} \right)/v_{\text{slo},m} \right)}$	(5)

$$\tau_{m}=\frac{1}{t_{adj}}\left( \tau_{m,min}+\frac{\tau_{m,diff}}{1+exp\left( -\left( v_{\text{off},m,t}-V_{m} \right)/v_{\text{slo},m,t} \right)} \right)$$

$h_{\infty}=\frac{1}{1+exp\left( \left( v_{\text{off},h}-V_{m} \right)/v_{\text{slo},h} \right)}$	(6)

$$\tau_{h}=\frac{1}{t_{adj}}\left( \tau_{h,min}+\frac{\tau_{h,diff}}{1+exp\left( -\left( v_{\text{off},h,t}-V_{m} \right)/v_{\text{slo},h,t} \right)} \right)$$

where $v_{\text{off},m}$ = -40.0 mV, $v_{\text{off},m,t}$ = -35.0 mV, $v_{\text{off},h}$ = -90.0 mV, $v_{\text{off},h,t}$ = -50.0 mV, $v_{\text{slo},m}$ = 6.0 mV, $v_{\text{slo},m,t}$ = 5.0 mV, $v_{\text{slo},h}$ = 6.4 mV, $v_{\text{slo},h,t}$ = 7.0 mV, $\tau_{m,min}$ = 5.0 ms, $\tau_{m,diff}$ = 20.0 ms, $\tau_{h,min}$ = 20.0 ms, and $\tau_{h,diff}$ = 50.0 ms. The reversal potential is dependent on the local intracellular Ca2+ concentration and is thus a dynamic variable — in our simulations it was typically around 100–130 mV but could temporarily reach values as low as 50 mV (data not shown). For the describption of the other current species, we refer to the original publications [Hay et al., 2011, Almog and Korngreen, 2014].

2. There is no explanation for the scale bar in figure 2 A, E, and F.

Thanks, we have now added this in the caption of the figure: “Scale bars 5 ms and 50 mV”.

3. Authors need to explain what the activation variables and the inactivation variables of the low-voltage activated calcium (LVA) channels are in the results 3.2.

We have now explained this in more detail in the main text and Figure captions and referred to the equations mentioned in our above comment. In Section 3.2, on p. 9 we now write:

As suggested by previous modelling work of CA1 neurons [Tsay et al., 2007], the reason behind the interaction of LVA Ca2+ currents and the Ih current was that in the presence of Ih currents, the LVA Ca2+ channels were highly inactivated (see Equations 4 and 6) at resting membrane potential (Fig. 3E). Namely, the inactivation variable h of the LVA currents measured 800 μm from the soma had a resting value of 0.05 in the presence of Ih currents and 0.49 in the absence of Ih currents (Fig. 3E), suggesting many times stronger resting-state LVA currents in the Ih blocked case and thus strongly facilitated Ca2+ spike generation compared to the control case.

4. Authors need to explain what observation in supplementary figure 3 indicates the disappearance of the shunting in the results 3.2.

We have added explanatory sentences in the figure caption (Fig. S4). We now write:

Note the significantly larger values of *h* in (F) compared to control (C).

and

The observation that the response in (H) reached (and went beyond) that of the Ih blocked neuron indicates that increase in dendritic spike magnitude caused by Ih blockage is due to altered levels of inactivation, not activation, of LVA Ca^2+^ channels.

We also added a panel to Figure 3 (panel D) to better highlight the effect of LVA Ca^2+^ channel conductance in the hot zone on the shunting. In Figure 3 caption we now write:

D: Threshold current amplitudes according to Hay model without Ih currents where the conductance of the LVA Ca2+ channels in the hot zone was gradually reduced toward the baseline LVA Ca2+ channel conductance in the apical dendrite (0% and 100% curves are identical to the corresponding curves in (B)).

5. How did authors calculate the factor by which the threshold conductance of the Ih-blocked is changed in figure 4-6? Authors need to mention this in the figure legend or methods.

We now clarify how the factor by which the threshold conductance changed in the caption of Figure 4:

These factors were calculated as fractions of the threshold conductances g_Threshold,Ihblocked_/g_Threshold,control_

We have now also mentioned how the threshold conductances were calculated in the Methods. In Section 2.1, on p. 4 we write:

Threshold currents and conductances were sought for using bisection method.

The authors only report firing rate changes when evaluating the effectiveness of HCN conductance and neuromodulators. This approach is reasonable because spike activity is the output from neurons. This study could be more informative and attract more interest if the authors provide detailed electrophysiological data (for example, the magnitude of stimulation evoked postsynaptic potentials and the resting membrane potentials). The authors would be able to easily collect these parameters because the NEURON simulator has current-clamp and voltage-clamp functions.

We thank the reviewer for this suggestion. We have now added voltage traces to our main figures that were missing them (Fig. 1, Fig. 4, Fig. 5, and Fig. 6), thus providing both resting-state membrane-potential and EPSP magnitude data at soma and dendrites. We also added Fig. S5 to provide membrane potential time courses for the original Almog model and the Almog model including a hot zone of LVA Ca^2+^ channels.

Reviewer #2: There are some very interesting points made in this paper about the distance dependent effects of Ih and about the interplay between Ih and Ca channels. This should be focus of the paper and there should be more information supporting what is known about Ca channel types and locations. All figures would benefit from addition of voltage traces.

We thank the reviewer for this comment. We have now added voltage traces to the figures that were missing them (Fig. 1, Fig. 4, Fig. 5, and Fig. 6). We have also extended the discussion by including a subsection on Ca^2+^ channels expressed in L5PCs. On p. 19, we now write:

4.2 Role of LVA Ca2+ channels and their interaction with I_h_ channel modulation in L5PCs

Our results consistently suggest that LVA Ca2+ currents are needed for the shunting effect of the Ih channels. LVA Ca2+ currents are mediated by T-type Ca2+ channels, which are functional as single α1 subunits (encoded by CACNA1G, CACNA1H, or CACNA1I, all of which are mRNA-expressed in mouse L5PCs [Christophe et al., 2005]). The membrane expression of these channels is also strongly modulated by the presence of auxiliary subunits α2δ, β, and γ, but the presence of these subunits does not seem to affect their electrophysiological properties [Dubel et al., 2004]. T-type Ca2+ channels were found to be expressed in both soma and dendrites of L5PCs [McKay et al., 2006], and LVA Ca2+ currents were observed in electrophysiological experiments of L5PCs in [Markram and Sakmann, 1994, Kampa et al., 2006] but not in [Almog et al., 2009]. Since L-type Ca2+ channels, which require auxiliary subunits to be functional, are also expressed and functional in L5PCs, the T-type Ca^2+^-channel α1 subunits may also interact with the auxiliary subunits to increase their membrane expression [Dubel et al., 2004] — however, the distribution and selectivity of the auxiliary Ca2+ channel subunits in L5PCs remains unknown. If future research reveals subclasses of thick-tufted L5PCs possessing different magnitudes of LVA Ca2+ currents (or substantially different distributions of LVA Ca2+ channels), our results will help to classify these groups into Ih-shunting and non-shunting subpopulations.

T-type Ca2+ channels are also regulated by neuromodulators. Dopamine, mediated by D1Rs, was found to inhibit T- type Ca2+ currents in glomerulosa cells from rat adrenal glands [Drolet et al., 1997], and similarly, acetylcholine inhibited Cav3.3-type LVA Ca2+ currents in HEK293 cells, mediated by co-transfected M1Rs [Hildebrand et al., 2007]. By contrast, mAChR agonist carbachol increased T-type Ca2+ currents in [Ohkuma et al., 2013]. Our results suggest that some of these observed effects could in fact be due to the effects of these neuromodulators on Ih currents, which, by altering the resting membrane potential, have downstream effects on the degree of inactivation of LVA Ca2+ channels. For example, even the HEK293 cells, which are widely used as a model system for studying transfected ion channels only, natively express mRNA encoding Ih channel subunits [Varghese et al., 2006]. We thus suggest that experimental settings studying the effects of neuromodulators on T-type Ca2+ channels should control the interaction of the Ih channels to prevent mixed effects of the two types of channels. Nevertheless, once data on the effects of neuromodulators on ion-channel activity are obtained for both T-type Ca2+ channels as well as other ion channels expressed in the L5PCs, these effects could be involved in our modelling framework to produce predictions of total effects — instead of Ih-mediated effects only as studied in our work — of neuromodulation on L5PCs.

Reviewer #3: * Major Comments
** The authors describe interesting differences in the effect of HCN channels depending
on the presence or absence of Ca2+ channle hot zones, but don't mention whether or not
their simulations support the existence of hot zones, or if they predict some L5PCs to
have hot zones and other don't, or if the Ca2+ channels in the hot zones may be themselves
blocked or modulated. It is interesting to note that in Almog & Korgreen (2014), they tried
adding a hot zone and couldn't achieve dendritic spikes. Does your altered version of the
Almog model with the Ca2+ channel hot-zone produce dendritic spikes?
ubiquity of Ca2+ channel hot-zones?

We thank the reviewer for his comments. Relatively little is known about the exact nature of the hot zones, and about how ubiquitous they are in L5PCs. Larkum et al. 1999, where the authors used electrophysiology and Ca^2+^ fluorescence imaging, showed that the largest peak Ca^2+^ concentration in response to a combination of somatic (intracellular electrode) and distal apical (extracellular electrode) stimuli was observed between 400-700 um from the soma in 10 out of 12 tested L5PCs, which suggests an over 80% prevalence of what later became known as hot zones. As reviewed in Almog and Korngreen 2014, further characterization of the Ca^2+^ spike generation in the distal apical dendrite were made in the nineties and 2000’s using similar tools. Surprisingly little attention has been paid in the past 10 years on this question despite the advances made in the experimental techniques. As far as we are aware of, apart from McKay et al. 2006 which we now refer to, no data on imaging of fluorescent labeled Ca^2+^ channels along the apical dendrite have been published. This may be due to the challenge of low fluorescence intensity in thin dendrites, but it could also be due to the complex relationship between the expression of Ca^2+^ channel subunits and the electrophysiological characteristics of Ca^2+^ spike generation at the apical dendrite. Namely, instead of encountering an overexpression of Ca^2+^ channel alpha-pores in the hot zone it may be that the alpha-pores of Ca^2+^ channels are equally distributed along the dendrites while the auxiliary Ca^2+^-channel subunits are more strongly expressed within the hot zone, that the Ca^2+^ channels undergo different post-translational modifications depending on their location, or that the presence of some other proteins inhibit the Ca^2+^ channels in dendritic regions other than the hot zone.

Of note, our models (likewise models of other people) assume the increased maximal Ca^2+^ conductance at the hot zone but do not make claims on what is its cause. However, future work should eventually go beyond purely electrophysiological evidence to look for the cause – this would help not only to improve the existing models (e.g. by fine-tuning the localization of the hot zone) but to introduce new model variables that can be differently modulated in the model (e.g., if the hot zone contains the same number of Ca^2+^ channels as the rest of the dendrite but they are differently phosphorylated, this may have consequences on how the neuromodulation affects Ca^2+^ channels in different parts of the dendrite). While our results suggest that whenever there are shunting effects of I_h_ currents in L5PCs there must be a hot zone of LVA Ca^2+^ channels, we believe that the prevalence and nature of the hot zones of Ca^2+^ channels in L5PCs should be verified by imaging (both Ca^2+^ fluorescence and Ca^2+^ channel labeling) and pharmaco-electrophysiological studies. Once that is done, our model can reveal whether and how much I_h_ channels shunt the inputs at the apical dendrite based on the strength of the LVA Ca^2+^ channels.

In Discussion, on p. 19 we now write:

Our results consistently suggest that LVA Ca2+ currents are needed for the shunting effect of the Ih channels. LVA Ca2+ currents are mediated by T-type Ca2+ channels, which are functional as single α1 subunits (encoded by CACNA1G, CACNA1H, or CACNA1I, all of which are mRNA-expressed in mouse L5PCs [Christophe et al., 2005]). The membrane expression of these channels is also strongly modulated by the presence of auxiliary subunits α2δ, β, and γ, but the presence of these subunits does not seem to affect their electrophysiological properties [Dubel et al., 2004]. T-type Ca2+ channels were found to be expressed in both soma and dendrites of L5PCs [McKay et al., 2006], and LVA Ca2+ currents were observed in electrophysiological experiments of L5PCs in [Markram and Sakmann, 1994, Kampa et al., 2006] but not in [Almog et al., 2009]. Since L-type Ca2+ channels, which require auxiliary subunits to be functional, are also expressed and functional in L5PCs, the T-type Ca^2+^-channel α1 subunits may also interact with the auxiliary subunits to increase their membrane expression [Dubel et al., 2004] — however, the distribution and selectivity of the auxiliary Ca2+ channel subunits in L5PCs remains unknown. If future research reveals subclasses of thick-tufted L5PCs possessing different magnitudes of LVA Ca2+ currents (or substantially different distributions of LVA Ca2+ channels), our results may help to classify these groups into Ih-shunting and non-shunting subpopulations.

As mentioned by the reviewer, Almog et al. 2014 suggested that a hot zone containing MVA Ca^2+^ channels was not excitable enough to generate dendritic Ca^2+^ spikes, and thus we used the LVA Ca^2+^ channels to begin with. The Almog model where this hot zone was added indeed produces Ca^2+^ spikes. This is now illustrated in Fig. S5. On p. 9, we write:

The Almog model implemented with the hot zone of LVA Ca2+ channels produced dendritic Ca2+ spikes that typically induced a burst of APs (Fig. S5), similar to the Hay model [Hay et al., 2011].

** The effects of HCN blockade, overexpression, and modulation are very small in the Hay model
compared to the Almog model (Fig. 1). The difference between the scales of the F-I curves and
bar graphs for the two models also, at a glance, serves to amplify the difference in the Hay model
relative to the Almog model. I would suggest either placing them on the same y-scale or
mentioning the differences in scale in the figure caption.
It would be very helpful to list either the change or percent change in threshold current
amplitudes for inducing an AP and/or the distance of the half-max values of the F-I curves in the
text to make this difference between the models more clear.

We have now mentioned the different scales in the caption of Figure caption and displayed the relative change of threshold currents in the insets of panels E-H. We also mention the effects of neuromodulation on the area under curve (AUC) of the f-I curves. In the caption of Figure 1, we now write:

The insets of panels (E)–(H) show the threshold current amplitudes for inducing an AP, and the relative changes to the threshold current amplitude of the control model are displayed next to the corresponding bars (the threshold current amplitudes can be different from the onsets of the f-I curves since some DC amplitudes only cause one spike). Note the difference in axes scales between the models in (E–H). Areas under curve, Almog model: 0.76 (Ih blocked), 3.1 (Ih modulated by acetylcholine), 6.0 (control), 11.9 (Ih overexpressed), and 13.1 (Ih modulated by dopamine) nA·Hz. Areas under curve, Hay model: 14.0 (Ih blocked), 14.8 (Ih modulated by acetylcholine), 15.2 (control), 15.6 (Ih overexpressed), 15.6 (Ih modulated by dopamine) nA·Hz.

** The difference in effects of HCN blockade, overexpression, and modulation also
begs the question: How different are the distributions of HCN
channels between these models, and how different are the models of Ih used in the two models
themselves. The original papers where the Ih models were developed (Kole, Hallermann,
and Stuart, J. Neurosci. 2006 and Williams and Stuart J. Neurophysiol 83:3177, 2000) should
be cited and a description of the differences between them included in the text.
Furthermore, a more detailed description or supplementary figure showing
the spatial distribution of Ih in these models (like plot of gbar for Ih vs. distance from soma)
might help in explaining this difference. Not only might the differences in HCN models and
distributions explain the differences between the models seen in Fig. 1, they might also
contribute to the effects of the Ca2+ hot zones described later on.
I noticed that the Ih models from each model neuron are copied into the other's folder
(e.g. modulhcn/modeulhcn_almog/IhHay.mod), but I'm curious if you ever tried swapping these
Ih models in the neurons and running even just the F-I curve protocol.

We thank the reviewer for this relevant point. We added the description of the two most central ion-channel currents in our models, namely, the I_h_ and the LVA Ca^2+^ channel currents. We also added the references mentioned by the reviewer when describing the activation kinetics and distribution of the I_h_ channels. In Methods, Section 2.1, on p. 3 we now write:

The dynamics of the I current, gated by the inactivation variable h as $I_{h}=\bar{g_{h}}h\left( E_{h}-V_{m} \right)$, is described as follows. In both models, the variable *h* obeys the equation

$$\frac{dh}{dt}=\frac{1}{\tau_{\infty}}\left( h_{\infty}-h \right)$$

In the Hay model, $h_{\infty}$and $\tau_{\infty}$are described as

$$\alpha_{h}=-\frac{v_{\text{off},a}-v}{\tau_{a}}\frac{1}{exp\left( -\left( v_{\text{off},a}-V_{m} \right)/v_{\text{slo}} \right)-1}$$

$$\beta_{h}=\frac{exp\left( -\left( v_{\text{off},b}-V_{m} \right)/v_{\text{slo},b} \right)}{\tau_{b}}$$

$h_{\infty}=\frac{\alpha_{h}}{\alpha_{h}+\beta_{h}}$	(1)

$$\tau_{\infty}=\frac{1}{\alpha_{h}+\beta_{h}}$$

with $v_{\text{off},a}$ = -154.9 mV, $v_{\text{slo},a}$ = 11.9 mV, $\tau_{a}$ = 155.521 ms, $v_{\text{off},b}$ = 0.0 mV, $v_{\text{slo},b}$ = 33.1 mV, and $\tau_{b}$= 5.18135 ms. In the Almog model, $h_{\infty}$and $\tau_{\infty}$are described as

$h_{\infty}=\frac{1}{1+exp\left( \left( V_{m}-v_{\text{off}} \right)/v_{\text{slo}} \right)}$	(2)

$$\tau_{\infty}=\frac{1}{t_{adj}\left( \frac{1}{t_{0}}exp\left( \left( v_{\text{off},t1}-V_{m} \right)/v_{\text{slo},t1} \right)+\frac{1}{t_{1}}exp\left( -\left( v_{\text{off},t2}-V_{m} \right)/v_{\text{slo},t2} \right) \right)}$$

$$t_{adj}=q_{10}^{\left( T-{22}^{\circ}\text{C} \right)/{10}^{\circ}\text{C}}$$

where $v_{\text{off}}$ = -154.9 mV, $v_{\text{slo}}$ = 6 mV, $t_{0}$ = 2542.5883549 ms, $t_{1}$ = 11.40250855 ms, $v_{\text{off},t1}$ = 0 mV, $v_{\text{off},t2}$ = 0 mV, $v_{\text{slo},t1}$ = 40.1606426 mV, $v_{\text{slo},t2}$ = 16.1290323 mV, $q_{1}0$ = 2.3, and $T$ = 34$^{\circ}$C. The reversal potential of the *I_h_* current was more depolarized in the Almog model (*E_h_* = -33 mV) than in the Hay model (*E_h_* = -45 mV).

We also added a figure (Fig. S1) to illustrate the spatial distribution of I_h_ channels; in Section 2.1, on p. 3 we write:

The distribution of the Ih channels in the two models are illustrated in Fig. S1.

Indeed, while exploring the cause for the difference between the results in the two models, we swapped certain ion-channel mechanisms between the two models. In particular, exchanging the I_h_ mechanisms between the two models did not affect the qualitative difference between them – the Almog model still showed that I_h_ current had an excitatory role and the Hay model still had a switch from excitatory to inhibitory effect around 700 um from the soma (see Fig. 2). We decided to leave these simulations out of the present manuscript as we already had many supplementary figures confirming our observations.

** The authors need to distinguish the neurons they are studying, thick tufted L5PCs with high HCN
channel expression that project to subcortical structures like pyramidal-tract neurons in motor cortex,
to thin tufted L5PCs which have low HCN channel expression and project elsewhere in cortex
(intratelencephalic).

We have now mentioned this more explicitly in the introduction. On p. 2, we write:

Unlike thin-tufted L5PCs that project across hemispheres through the corpus callosum, thick-tufted L5PCs express strong Ih currents and mostly project to subcortical structures [Hübener and Bolz, 1988, Le Bé et al., 2007].

* Minor comments
** The figure captions do a thorough job describing the contents of the figures but do
little to explain the results. Especially for the more complicated figures (Figs. 4-6),
some interpretation of the important results in the figure caption would help with readability.

We have now added sentences that help the interpretation of the results. In the caption of Fig. 4, we write:

“The threshold conductance was always larger in the absence of Ih channels.” (panels B-E)

“The threshold conductance was always larger in the absence of Ih channels (red squares), indicating a weak shunting effect of the Ih channels or no shunting at all.” (panel F)

“The threshold conductance was larger in the absence of Ih channels for proximal inputs (red squares) but smaller for distal inputs (blue squares), indicating a strong shunting effect of the Ih channels in the distal apical dendrite.” (panel G)

In the caption of Fig. 5 we now write:

”As in Fig. 4G, the threshold conductance was larger in the absence of Ih channels for proximal inputs (red squares) but smaller for distal inputs (blue squares). This figure shows that the frontier between shunting and excitatory effects of the Ih currents was pushed further or brought closer by simultaneous excitation or inhibition, respectively, at the basal dendrite.” (panel A-B)

In the caption of Fig. 6 we write:

The upward triangles of panel (C) largely overlap the downward triangles of panel (D) at the distal parts of the apical dendrite, suggesting that for distal inputs the effects of proximal dopamine and distal acetylcholine are cumulative, and likewise, the effects of proximal acetylcholine and distal dopamine are cumulative. By contrast, the mixed blue and red squares for stimuli reaching proximal dendrites indicate compensatory effects of proximal dopamine and distal acetylcholine, and vice versa.

** Figure three is hard to follow (partly for the same reasons as above). Recommend
including "dim" curves in the figure legend (like control and block Ih) or use
different colors/linestyles; otherwise I needed to keep going back and forth between
the figure, the caption, a previouf figure, and the legend,

We thank the reviewer for his comments that help us improve the clarity of the manuscript. We have done the suggested changes.

** [page 6] The phrase "increased I h activation by lamotrigine application" makes it sound like
lamotrigine is an HCN channel agonist. Please clarify.

Indeed, lamotrigine serves as a potentiator (some researchers even call it an agonist, see Omrani, Azar, et al. *Molecular psychiatry* 20.11 (2015): 1311-1321) of HCN channels. We now refer to it as an “enhancer” of HCN channels.

** The descriptions of the synaptic stimulation protocols should be outlined in the Methods section;
whereas now they are really only described in detail in the figure legends.

We have now included the information on synaptic stimulation protocols in the section Stimulation protocols of the Methods, Section 2.2. On p. 5, we write:

In simulations containing the AMPA- and NMDA-receptor or GABA-receptor synapses, we distributed 2000 simultaneously activated synapses across the apical or basal dendrite, typically restricting to distances [x1,x2] from the soma, where the parameters x1 (x-axis) and x2 (y-axis) ranged from 200 μm to 1300 μm in intervals or 100 μm.

** [page 18] It's worth noting that one of the "range of neocortical L5PC models" examined in Kelley et al. (2021)
was the Hay model, which did not fit the experimental phase data, but that probably does not have
much influence on the phenomena the authors describe. Furthermore, "our models
suggest that when using a realistically morphological model of an L5PC" implies that the models used in
Migliore & Migliore (2012) and Kelley et al. (2021) were not morphologially realistic or detailed, which
they were.

We agree that the qualifier “realistically morphological” creates an unnecessary contrast in this sentence, thus we have removed it. We also mention that the Hay model was considered in the paper. The sentences now read:

[Kelley et al., 2021] examined a range of neocortical L5PC models (including the Hay model), and suggested that a coupling of TASK-like channel with the Ih currents as delineated in [Migliore and Migliore, 2012] provided the best fit to experimental impedance amplitude and phase data. While it is possible that different mechanisms modulate Ih influence on neuron excitability on different regions, our models suggest that T-type Ca2+ channels and not M-type K+ channels have a role in the shunting or excitatory effects of Ih currents.

** [page 18] "Thus, the apical inputs act as the context that exerts a modulation over the content that arrives
to the basal input". Please carlify how apical inputs represent "context" while basal inputs represent
"content".

This is the classical higher-level understanding of the role of L5PC, that the inputs arriving to apical dendrite (mostly from higher-order thalamic and cortical regions) convey information about the “context” through the feedback circuitry whereas the inputs arriving to basal dendrites (mostly from lower-order thalamic and sensory areas) encode the “sensory content”. We have now rewritten these sentences to better convey this message. In Discussion, Section 4.4, on p. 20 we write:

Inputs to the basal dendrite or perisomatic zones are related to the preferred sensory stimulus of the neuron, that is, the information itself that the neuron transmits as part of a feedforward circuit. These inputs can be thus referred to as the specific “content” that a L5PC neuron encodes. On the other hand, inputs to the apical dendrite arrive from higher-order thalamus, feedback loops from prefrontal cortex, subcortical structures such as amygdala, and neuromodulatory regions [Gur and Snodderly, 2008, Rubio-Garrido et al., 2009]. The information contained in these inputs encodes arousal or vigilance state, goal information, or state predictions [Arnsten, 1998]. These apical inputs modulate the strength of the effect that the input to the somatic compartment has on the neuron’s excitability, without changing the preference between different stimuli. Thus, the apical inputs act as the “context” that exerts a modulation over the content that arrives to the basal input.

We also adapted the sentence on p. 1-2 of the introduction as follows:

Thanks to their complex morphology and connectivity, feed-forward sensory- related stimuli and feed-back context-dependent inputs arrive at spatially distinct sections of the L5PC dendritic tree, the former stimulating the basal dendrites and the latter largely exerting their action on the distal apical dendrite [Larkum, 2013].
