# Supplementary material for: Excitatory and inhibitory effects of HCN channel modulation on excitability of layer V pyramidal cells
Source: PLoS Comput Biol. 2022 Sep 13;18(9):e1010506. doi: 10.1371/journal.pcbi.1010506 (PMC9506642; doi:10.1371/journal.pcbi.1010506)
Supplement: Attachment — Submitted filename: reply_to_reviewers_thirdsubmission.docx [file pcbi.1010506.s015.docx]

Dear Editors,

We are resubmitting our manuscript “Excitatory and inhibitory effects of HCN channel modulation on excitability of layer V pyramidal cells”. In response to the comments by Reviewer 1, we have carefully examined the literature to find out whether our models of neuromodulation of I_h_ channels in pyramidal cells are physiologically realistic. While we agree with the reviewer that more data from cholinergic and serotonergic modulation may be needed to confirm the suggested models of Ih modulation through these pathways, we found data strongly suggesting that catecholamines (dopamine and noradrenaline) modulate I_h_ channels in pyramidal cells in the described manner. Due to the bimodal effects of most neuromodulators through different receptors and, in many cases, verified effects of both modalities in cortical pyramidal cells, we now refer to the modelled neuromodulation as “cAMP-enhancing” and “cAMP-inhibiting” modulation instead of “dopaminergic” and “cholinergic” – this also serves to keep the scope of our article more strictly in the I_h_ channels instead of having to discuss the electrophysiology of pyramidal cells at large in response to these two formerly highlighted neuromodulators. To assure the readers that our models are physiologically realistic, we now show that the effects of a -10 mV shift employed in the Hay model as a model of cAMP-inhibiting neuromodulation closely resemble those observed in an experimental paper employing a physiologically realistic concentration of dopamine. We believe the manuscript is improved in consequence of these changes. Please see below for our point-by-point addressing of the reviewers’ comments (reviewers’ comments in red, our reply in black, and quotations of the revised manuscript in blue).

On behalf of the authors,

Tuomo Mäki-Marttunen

The authors appropriately and convincingly addressed the concern of different cAMP sensitivities between HCN1 and HCN2. Because no experimental study has empirically evaluated the in vivo ratio of HCN1/HCN2 in L5PCs, the authors addressed this concern by testing an extreme condition of strictly HCN1 channels in their model. The authors added a new result with minor voltage dependency shift reflecting the lower cAMP sensitivity of HCN1. The authors also address the possibility for heteromultimeric channels, the presence of which would not be expected to change the crux of their results.

However, I also noted that HCN1 and HCN2 differ not only in their sensitivity to cAMP, but also their activation speed (see figure 1B in Chen et al., 2001, and figure 1B in Ulens and Tytgat, 2001, both papers are already in the reference list). HCN1 channels activate several times faster than HCN2 channels. This difference potentially affects the spike generation in the short time window that is tested in many simulations of this study. Therefore, the authors need to adjust the channel kinetics for heteromeric HCN channels at a minimum (or show that all results are consistent with the results in HCN1 only condition, which represents a greater extreme) in addition to the cAMP sensitivity.

We thank the reviewer for this comment. The time constant of the modeled Ih channels was typically (within subthreshold membrane potentials) in the range 50-100 ms (see Fig. 5A of Kole et al. 2006, which the Ih current of the Hay model is based on, and Fig. 1F of Williams and Stuart 2000, which the Ih current of the Almog model is based on). This is in good agreement with the time constants of the HCN1/HCN2 channels measured in Ulens and Tytgat 2000 ($\tau_{fast}=99.8 \mathrm{ms},\tau_{slow}=440 ms$). As for HCN1-only channels, Ulens and Tytgat 2000 reported a 3- to 4-fold faster time constants ($\tau_{fast}=23.7 \mathrm{ms},\tau_{slow}=138 ms$). We thus implemented the HCN1-only channels with 4-fold faster activation/deactivation dynamics. The results remained qualitatively unaltered – we now report these results too (Fig. S8A-D). We also confirmed that the threshold currents of the short pulses injected at the apical dendrite were unaffected by the shorter time constant (Fig. S8E).

On p. 12, we now write:

The statistical significance of these results remained even if we assumed a weaker, HCN1-like modulation, both when a normal (Fig. S7) and faster (Fig. S8A-D) time constant of Ih inactivation was used.

On p. 23 we write:

Our predictions for the effects of neuromodulation (Fig. S8A–D) and blockage (Fig. S8E) of Ih channels on L5PC excitability were also unaffected if a smaller time constant, typical to homomeric HCN1 channels, was used.

I originally pointed out the discrepancy in the neurotransmitter dose between physiological and experimental conditions (and the potential gap in the electrophysiological effect between them). Note that the experimental conditions are not necessarily physiological conditions. Experiments performed in vitro (i.e. patch clamp recordings in slice) do not represent a physiological condition that can be studied in vivo (i.e. microdialysis in live animal cortex). For example, Hironaka et al. (2001) recorded ***in vivo*** Ach concentrations, while Zhao et al. (2016) examined HCN channel properties ***in vitro*** using a far larger dose of Ach in the extracellular solution than ever observed in a live animal. Therefore, I noted that it was not appropriate for the authors to use a similar degree of voltage shift reported by Zhao et al. 2016.

In the authors’ response, the authors referenced the similar magnitudes of voltage shifts between two experimental conditions (extracellular application of agonist and intracellular perfusion or inside-out patch clamp). However, they did not show if the dose of neurotransmitters in such studies are physiological. Therefore, the authors did not address my concern. If the authors intend to simulate the physiological condition, parameters must be within the physiologically realistic range.

We agree with the reviewer that it is important to model physiologically realistic rather than unrealistic events. After another round of literature review, we have made significant changes to the manuscript concerning the hypothesized origins of HCN channel modulation in L5PCs. The underlying neuromodulators (especially dopamine) have been shown to modulate ion channels in pyramidal cells with relatively low, physiologically realistic concentrations. Below, we argue for the choices we made in respect to each of the concerns raised by the reviewer.

I expressed the concern that the authors' simulations used a degree of voltage shift obtained from experiments in which the dose of neurotransmitter was more than 200 times higher than the physiological dose.

To address this concern, the authors explained that the dose of neurotransmitters inside the synaptic cleft is higher than in the extracellular space. This idea is reasonable if the neurotransmitter is released and the receptor for the same neurotransmitter exists in the same synapse. However, this model is often not applicable to modulatory neurotransmitters and their receptors. The authors should refer to work on the synapses and axon terminals of the pyramidal neurons as addressed by electron microscopy (see below).

In the case of M2-type muscarinic receptor, Mrzljak et al. reported that the M2 receptors localize exclusively at asymmetric synapses in pyramidal neurons (Mrzljak et al., 1998, PMID: 9456180). However, cholinergic synapses in pyramidal neurons are not asymmetric synapses (Mrzljak et al., 1995, PMID: 7673486). Therefore, most M2 receptors do not receive the acetylcholine released in the same synapse. These observations indicate that cholinergic neurotransmission to M2 receptors in pyramidal neurons occurs via volume neurotransmission. Dopamine is released in a similar manner, and the D1 receptor is also not localized to the dopaminergic terminal (Smiley et al., 1994, PMID: 7911245).

Therefore, we cannot expect a high dose of acetylcholine and dopamine in the M2 receptor-positive and D1 receptor-positive synaptic clefts in the cortical pyramidal neuron. The authors need to use the extra- synaptic dose for both acetylcholine and dopamine and adjust the degree of voltage shift in the model accordingly. In my first comment I linked papers that directly measure the extracellular concentrations for acetylcholine and a theoretical model estimating dopamine concentrations. I expect the authors to repeat their simulations using these estimates, which are, to my knowledge, our best guess of the physiological operating range of these two neuromodulators. If the authors know of another reference for in vivo extracellular (i.e. physiological) concentrations then I could be convinced of a higher dose.

We thank the reviewer for these references and discussion on cholinergic and dopaminergic neurotransmission. A review by Sarter et al. 2009 (PMID: 19377503) examines many observations in favor and against cholinergic transmission occurring through volume transmission and concludes that the prevalence of volume transmission of ACh over wired transmission remains an unresolved issue. To highlight a remark made in this review, we point out that even if activation of M2 receptors happens through volume transmission, the distance between neurotransmitter ejection site and receptor may be shorter than thought since a large fraction of ACh can be released non-synaptically as suggested by Vizi and Lendvai 1999 (PMID: **10567725**). It should also be noted that Mrzljak et al. did not label the M4 receptors which are expressed in cortical pyramidal neurons (Amar et al. 2010, PMID: **20600670**) and, similar to M2 receptors, are coupled to Gi/o thereby decreasing cAMP. However, we agree with the reviewer that there is little evidence of the effects of M2-type receptor activation on ion-channel activity in the cortex. Thus, we see that it is best not to use M2 receptor activation as the primary example of cAMP-decreasing neuromodulation of Ih channels in pyramidal cells since there is stronger support for the effects of other cAMP-decreasing neuromodulators (see below).

The reviewer suggested that we adjust the model to data from experiments using lower neuromodulator concentrations, such as those observed in Hironaka et al. (PMID: 11368961) or predicted in Spühler and Hauri 2013 (PMID: 23951205). While we are positive toward using data directly from experiments, we are cautious about the neuromodulator concentration ranges reported in these two papers for the following reasons.

Hironaka et al. measured the concentrations using microdialysis recording. Although this is probably the most accurate method, it suffers from an extremely low spatiotemporal resolution. In Hironaka et al., a single recording tube (220 um diameter) per brain area was used, and although the length of the recording period was not mentioned, it was most likely a few minutes (not more than 10 min because the experiment was repeated every 20 min with 10 min waiting periods). Hironaka et al. observed significantly increased ACh concentration during the behavioral period of the delayed alternation task or its variant in PFC and hippocampus (30 nM) compared to the pre-behavioral period (10-20 nM). Slightly larger values (60-120 nM) were observed in Ihalainen et al. 2010 using similar microdialysis technology. However, it is reasonable to believe that these grand-average neurotransmitter concentrations (averaged across both space and time) are orders of magnitude smaller than the local, transient concentrations following neurotransmitter release. Firstly, Parikh et al. 2007 (PMID: 17920021), who used (temporally) high-resolution amperometry, measured transient (lasting seconds) choline concentrations reaching 2-4 uM in PFC – the validity of choline measurement in place of acetylcholine was confirmed in the earlier papers of the authors. Secondly, the neuromodulator concentration varies spatially, and the large diameter of the recording tubes prevents more detailed data from being collected. Wiencke et al. 2020 (PMID: 33253315) show through computational modelling that due to diffusion the peak neuromodulator concentration can decrease 10-fold within 1-2 um distance and 100-fold within 2-4 um distance. This implies a huge spatial variability in the concentration of the neuromodulator. Furthermore, note that some researchers question the reliability of microdialysis for determining absolute neurotransmitter levels altogether although they laud its potential in reporting relative changes (Chefer et al. 2009, PMID: 19340812).

The issues of both temporal and spatial resolution are avoided by the computational modelling of Spühler and Hauri 2013 referred to by the reviewer. In Spühler and Hauri, the DA concentration increased from 100 to 200-300 nM on average in the vicinity (at 1 um distance) of the DA source. Although it was reported that the steady-state concentration could reach values as high as 700-1000 nM following phasic stimulation in their simulations, the predicted values of dopamine concentrations in Spühler and Hauri may still be underestimated. This is because a relatively sparse density of dopamine release sites (200’000/mm^3^) was assumed in Spühler and Hauri. Vitrac et al. 2014 (PMID: 24616667) estimate the density of dopaminergic terminals to be 1’380’000/mm^3^ in motor cortex. Audet et al. 1988 (PMID: 2464617) estimate the density of noradrenergic terminals to be around 1’200’000/mm^3^ in all measured cortical areas (including prefrontal cortex), and while the density of dopaminergic terminals was typically smaller than that of noradrenergic terminals, in mPFC the numbers were comparable. Furthermore, note that dopamine is co-released from noradrenergic terminals in the cortex (reviewed in Ranjbar-Slamloo and Fazlali 2020, PMID: 32038164) – thus it would not be unrealistic to expect up to 10 times larger extracellular dopamine concentrations. The spatial heterogeneity of the dopamine concentration may also be underestimated in Spühler et al. because a uniform dopamine uptake model was used instead of a more physiologically realistic one where the dopamine transporters would only be distributed along the axons of dopaminergic neurons.

We thus believe that neuromodulator concentrations can rise much higher than 1 uM outside the synaptic cleft. However, we agree with the reviewer that 200 uM bath-application of dopamine in Byczkowicz et al. is likely to be exaggerated – the authors probably chose a concentration that for sure saturates the dopamine receptors. Gulledge and Jaffe 1998 examined the effects of varying dopamine concentration on L5PC excitability (mediated by the higher-affinity D2 receptors): while 10 nM showed no effects, similar D2R-mediated excitability-decreasing effects were observed for 50, 100, 500, 1000, and 3000 nM. Importantly, we have now showed that the effects of our -10 mV shift of the Ih half-inactivation curve in the Hay model closely resembled the effects of 500 nM bath-applied dopamine in their paper. Furthermore, D1R activation has been documented for smaller dopamine concentrations in layer V-VI pyramidal cells: Maurice et al. 2001 (PMID: 11264302) showed that D1/D5R activation by 50 uM dopamine reduced fast Na+ currents, and Lavin and Grace 2001 (PMID: 11377838) showed that D1R activation by 30 uM dopamine increased the resting membrane potential and neuronal excitability. In layer 2/3 pyramidal cells of ACC, extremely large effects on half-inactivation voltage were observed for 50 uM dopamine (Lançon et al. 2021, PMID 34852233). Evidence exists that small micromolar, 5 uM, dopamine can also enhance Ih currents, although in a completely different system – the pyloric circuit (Rodgers et al. 2011, PMID: 22072689). Rodgers et al. also observed effects for nanomolar dopamine concentrations (5 nM), but in our opinion, a larger sample size would be needed to confirm this observation. Moreover, there are a few papers showing the activation of dopamine receptors in pyramidal cells using a combination of electrical stimulation and dopamine receptor antagonists (i.e., without bath-application of dopamine or the corresponding agonists; Huang et al. 2004, PMID: **14981263; Lewis and O’Donnell,** PMID: 11073866). In addition to these there are the data on experiments with D1R agonists showing the coupling with Ih currents in mammalian L5PCs (e.g., Lancon et al. 2021, PMID: 34852233) – these are recent papers published in respectable journals by prominent researchers in the field, and we are inclined to believe that their findings reflect actual brain physiology although they use receptor agonists or somewhat larger neurotransmitter concentrations than expected. Taken together, we consider that there is enough evidence in favor of D1R and D2R activation with physiologically realistic dopamine concentrations in cortical pyramidal cells and their downstream effects on Ih channel activation.

In a similar manner, the 50 uM ACh used in Zhao et al. 2016 may be larger than necessary. Effects of different ACh concentrations on field potentials in visual cortex were studied in Kuczewski et al. 2005 (PMID: 15919709). The authors found that a 200 uM concentration of ACh activated M2/M4 receptors and led to a facilitation of field potentials whereas a yet larger concentration (1 mM) additionally activated M1/M3/M5 receptors and led to a depression of field potentials. The facilitatory effects were found the same for smaller concentrations (20, 50, and 100 uM). Given the suggested spatially averaged 2-4 uM acetylcholine concentration discussed above (Parikh et al. 2007), we believe that ACh levels can reach a concentration adequate for M2R activation at and near the spots of vesicle release.

How large then are the voltage shifts of HCN channels given physiologically realistic neuromodulator concentrations? This is a question we cannot answer accurately, but we can show that the estimates we gave are most likely in the correct range. DiFrancesco and Tortora 1991 (PMID: 1709448) studied the cAMP-dependency of Ih currents in oocytes and showed that while 10 uM cAMP gave a saturation of the voltage shift of Ih (17 mV for HCN2, 14 mV for heterotetrameric HCN1/HCN2, and 4 mV for HCN2) and 1 uM cAMP a close-to-saturation, even 0.3 uM cAMP produced a significant depolarizing shift (13-14 mV for HCN2, 9 mV for heterotetrameric HCN1/HCN2, and 3 mV for HCN1).

What are then the cAMP levels in response to catecholaminergic inputs in pyramidal cells *in vivo*? Measuring absolute cAMP concentrations along dendrites is extremely difficult but attempts to do this via computational modelling have been made. Ohadi et al. 2019 (PMID: 31668747) estimate that cAMP can reach 1-3 uM in a dendritic spine in presence of Ca2+ inputs and mild adrenergic receptor activation, depending on the localization of the adenylyl cyclases and phosphodiesterases. This would correspond to almost the maximal magnitudes of half-inactivation voltage shifts in the Ih channel according to the data of DiFrancesco and Tortora. A model such as this could be used to produce estimates of the cAMP concentration in rest and under catecholaminergic stimulation, but it would be out of the scope of our paper where the focus is anyway on qualitative (does cAMP-increasing/decreasing neuromodulation increase or decrease the L5PC excitability and under which conditions?) rather than quantitative questions.

Based on the discussion above, we argue that our estimate of 5-10 mV voltage shifts, which correspond to 29-71% of the maximal shifts in HCN1/HCN2 heteromers or HCN2 homomers are likely to be in the correct range of neuromodulation-mediated voltage shifts in L5PCs. We also point out that previous modelling studies have used these and larger values as models of neuromodulation: In Kase et al. (PMID: 22934165) $\pm$10 mV shifts were as a generic model of neuromodulation of Ih channels, and in Labarrera et al. (PMID: 29694883) a -15 mV shift was used as a model for strong (probably saturating) alpha2a-receptor activation-mediated modulation of Ih channels.

To discuss all these uncertainties, we added the following paragraph in the discussion (p. 23-24) in a subsection that we have now entitled “Notes on the magnitude of neuromodulation-mediated voltage shifts of Ih inactivation”:

The shifts of the Ih half-inactivation voltage used in our model may be over- or underestimated also due to the uncertainty about the strength of the activation of different neuromodulatory systems *in vivo* in L5PCs. Most of the experimental work addressing this question *in vitro* used bath application of neuromodulators or agonists of neuromodulatory receptors, which may cause stronger or weaker cAMP production or inhibition than what happens in endogenous neuromodulation *in vivo*. The neuromodulatory systems considered in this work have been suggested to operate through volume transmission rather than wired transmission [Smiley et al., 1994, Mrzljak et al., 1998, Quentin et al., 2018, Seguela et al., 1990] (note, though, that this view has been challenged in [Sarter et al., 2009]), and if this is the case, it is unsure whether the neuromodulator concentrations in the vicinity of the neuromodulatory receptors are large enough to significantly modulate the Ih channels. It has been estimated that volume-transmitted neuromodulators can locally reach micromolar concentrations. Namely, maximal acetylcholine concentrations of 2-5 μM were measured by amperometry in the PFC [Parikh et al., 2007], and a computational modelling study estimated that extracellular dopamine concentration in the PFC can reach 250–1000 nM upon stimulation of dopaminergic axons [Spühler and Hauri, 2013]. As for noradrenaline and serotonin, their concentrations are expected to reach levels comparable to or higher than that of dopamine since the average levels of these neuromodulators are higher in the cortex [Versteeg et al., 1976, Dalley et al., 2002]. It should be noted that the estimates of dopamine concentrations made in [Spühler and Hauri, 2013] may be underestimated since they were based on an assumption of 200’000 release sites/mm3 while [Audet et al., 1988] suggests a density of approximately 1’200’000 release sites/mm3 in the PFC for noradrenaline and a comparable number for dopamine (noradrenergic terminals have later been reported to co-release dopamine *in vivo*, see [Ranjbar-Slamloo and Fazlali, 2020] for a review). Although these estimates of neuromodulator concentrations are lower than some of the bath concentrations used in the *in vitro* studies of Ih channels, the modulation of Ih has been confirmed with physiologically realistic neuromodulator concentrations as well. For example, a modest dopamine concentration of 500 nM caused a significant D2-mediated decrease in L5PC perisomatic excitability [Gulledge and Jaffe, 1998]. As for the lower-affinity D1 receptors, small micromolar (5 μM) dopamine concentration was found to alter the lateral pyloric excitability through increased Ih activity [Rodgers et al., 2011]. Also a relatively small concentration (10 μM) of serotonin significantly altered a subpopulation of L5PCs in rodent PFC [Elliott et al., 2018]. Importantly, activation of neuromodulatory receptors in L5PCs in response to endogenous neuromodulation and their effects on cellular or network electrophysiology have also been confirmed using antagonists of the underlying receptors. Namely, in [Huang et al., 2004], D1-receptor antagonism impaired an LTP in layer V of PFC caused by high-frequency stimulation of layer II in presence of picrotoxin, suggesting a non-assisted activation of D1 receptors and their contribution to synaptic plasticity in pyramidal cells. In another study, direct stimulation of the VTA *in vivo* in rats resulted in transitions to up-states in prefrontal cortical L5PCs in a manner indicating monosynaptic effects, and the duration of these up-states was significantly shortened by D1 antagonism [Lewis and O’Donnell, 2000]. Importantly, we replicated the effects of a physiologically realistic concentration (500 nM) on perisomatic excitability with an Ih-current half-inactivation voltage shift of -10 mV using the Hay model (Fig. S2E–F). Although more research is needed to map the employed neuromodulator concentrations to the measured intracellular cAMP concentrations and further to Ih voltage-dependence, this result suggests that our range of half-inactivation voltage shifts is likely to be in the correct order of magnitude.

The authors added a description of the serotonergic modulation of the HCN1 channel.

For the serotonergic transmission, Séguéla et al. found that only 38% of serotonergic varicosities formed a synaptic structure on dendrites and spines in the multiple cerebral cortices, and the remaining varicosities are nonjunctional (Séguéla et al., 1989, PMID: 2808757). Later, Smiley and Goldman-Rakic reconstructed the serotonergic synapses in the prefrontal cortex and reported that 92% of serotonergic synapses were made on the GABAergic interneuron and only 8% of synapses were on the dendritic shafts that showed features of pyramidal neurons (Smiley and Goldman-Rakic, 1996, PMID: 8698902). Therefore, pyramidal neurons have some serotonergic terminals on the dendritic shaft, although they are not major targets of serotonergic projection. However, DeFelipe et al. showed the intense exclusive labeling of 5-HT 1a receptors at the soma-axon compartment of the pyramidal neuron, and, in most labeled neurons, only the axons were labeled (DeFelipe et al., 2001, PMID: 11283956).

This discrepancy between the location of serotonergic synapses found on the dendritic shaft and the location of the receptor at the soma-axon shows that serotonergic transmission to pyramidal neurons is in a **paracrine** manner. As such, we would not expect to see a high concentration of serotonin gathered in a synaptic cleft. Therefore, without experimental evidence or a convincing computational study, 10 μM of serotonin in the external solution should not be considered a physiological condition.

We thank the reviewer for these references – we have now included references to Séguéla’s and DeFelipe’s work as evidence in favor of serotonergic neurotransmission occurring via volume transmission (see our reply above). There seems to be less estimates of serotonin concentration in the cortex under different scenarios than those of dopamine concentration, but some conclusions can be drawn by comparing data on serotonin and dopamine. Staiti et al. 2011 (PMID: 21527264) estimate (by microdialysis) that baseline 5-HT levels in the PFC are slightly larger than those of dopamine in almost all animal groups, and that under methamphetamine stimulation concentration (again measured by microdialysis) of both neuromodulators increase manifold (3-12 fold increase of 5-HT, 2-15 fold increase of dopamine). This provides evidence in favor of levels of serotonin being comparable to or higher than those of dopamine in both baseline and neuromodulated states, which is in line with the higher number of axonal serotonin terminals in the cortex (5’800’000/mm^3^; Descarries et al. 1990, PMID: 2252339) compared dopaminergic ones (Vitrac et al. 2014, Audet et al. 1988). Furthermore, the points we made above on the spatiotemporal fluctuations apply to serotonin as well. We thus believe the extracellular serotonin concentration, similar to that of dopamine, can rise to micromolar concentrations. The clearest evidence in favor of serotonin concentrations being high enough to modulate L5PCs came from studies directly stimulating the dorsal raphe. Activation of serotonergic fibers connecting to PFC resulted in an inhibition of pyramidal cell firing that is suggested to depend partly on GABAergic connections and partly on 5-HT1 receptors located in the perisomatic area of pyramidal cells (Puig and Gulledge 2011, PMID: 22076606). However, we agree with the reviewer that there are still gaps in our knowledge in respect to serotonergic and cholinergic modulation of the Ih channels in pyramidal cells that should be filled by future studies, and we state this out now more clearly in the discussion (see our reply above). Since the current evidence is stronger for noradrenergic alpha2a and dopaminergic D2-receptor-mediated downregulation of Ih currents in pyramidal cells, and since most neuromodulators have bimodal effects on cAMP levels through different receptors, we now refer to the neuromodulation as “cAMP-enhancing” and “cAMP-inhibiting” modulation instead of “dopaminergic” and “cholinergic”. To facilitate making connections between different neuromodulators mediated by different receptors and the effects on cAMP (enhancing or inhibiting), we have added a table in the introduction that summarizes the neuromodulation taking place in L5PCs as previously discussed in the literature. We now write in Introduction (p. 2):

We also modelled the response of the neuron when the Ih channels were under neuromodulation. Ih channels are bound to neuromodulatory effects through the cAMP intracellular pathway. cAMP binds to an Ih channel and increases its open probability. We examined the effect of cAMP-enhancing or cAMP-inhibiting neuromodulators (Table 1) by introducing experimentally observed effects of these neuromodulators on the voltage-dependence profile of Ih channels.

In Methods (p. 6), we write:

cAMP-enhancing and cAMP-inhibiting neuromodulation of Ih: We modelled the cAMP-enhancing modulation of Ih by increasing the half-inactivation voltage by +10 mV or +5 mV in the Hay or Almog model, respectively, and the cAMP-inhibiting modulation of Ih by decreasing it by the same amount. See Table 1 for the neuromodulator receptors mediating these effects. We used the smaller voltage difference (±5 mV) for the Almog model due to the relatively large effect of ±10 mV shifts (Fig. S12C). To model weakly cAMP-dependent Ih channels (HCN1 homomeric channels) in Fig. S7–S8, we used smaller half-inactivation voltage shifts, namely ±4 mV and ±2 mV in Hay and Almog models, respectively.

To mention the studies that report effects of serotonin on L5PCs, mediated by Ih channels, we now write on p. 18:

Moreover, similar to cholinergic M2 receptors, serotonergic 5-HT1A receptors (Gi/o-coupled) shift the half-inactivation voltage of Ih currents toward hyperpolarized potentials [Ko et al., 2016], and these receptors mediated an inhibitory net effect in a subgroup of prefrontal cortical L5PCs [Avesar and Gulledge, 2012, Elliott et al., 2018]. By contrast, activation of serotonergic 5-HT7A receptors (Gs-coupled) shift the half-inactivation toward hyperpolarized potentials [Hawkins et al., 2015], and this has been shown to contribute to depolarization of L5PCs in young rats [Beïque et al., 2004] and cats [Spain, 1994].

We also mention other sources of uncertainties on p. 19-20:

Fully addressing the question of neuromodulators’ actions on Ih-channel activity is curbed by the age-dependent and inter-species differences in expression of neuromodulatory receptors. For example, expression of serotonergic 5-HT7 receptors are highly expressed at birth but decreases during postnatal development [Muneoka and Takigawa, 2003], and 5-HT1 receptors were found to be expressed in the basal dendrites of rat L5PCs [Kia et al., 1996] while in primates they were found to be expressed mostly in axons [DeFelipe et al., 2001].
