# Supplementary material for: Excitatory and inhibitory effects of HCN channel modulation on excitability of layer V pyramidal cells
Source: PLoS Comput Biol. 2022 Sep 13;18(9):e1010506. doi: 10.1371/journal.pcbi.1010506 (PMC9506642; doi:10.1371/journal.pcbi.1010506)
Supplement: Attachment — Submitted filename: reply_to_reviewers_fourthsubmission.docx [file pcbi.1010506.s016.docx]

Dear Editors,

We are resubmitting our manuscript “Excitatory and inhibitory effects of HCN channel modulation on excitability of layer V pyramidal cells”. We have now made the minor revision suggested by the reviewer. Please see below for our point-by-point addressing of the reviewers’ comments (reviewers’ comments in red, our reply in black, and quotations of the revised manuscript in blue).

On behalf of the authors,

Tuomo Mäki-Marttunen

The authors pointed out that the density of the dopamine release site in their referenced study (Spühler and Hauri, 2013) is underestimated because Vitrac et al. 2014 reported a higher density than the reference. However, what Vitrac reported in their study is not the dopamine release site, but rather the mean length of the dopaminergic fiber per brain tissue volume (meter / mm^3). This is different from the density of the dopamine release site. The dopamine release site density was reported in the study (Table 2 in Descarries et al., 1987, PMID: 3627435) utilizing the same method as the newly added reference for the noradrenergic terminal density in the revised manuscript (Audet et al. 1998). Unless the authors specifically want to use the medial part of the frontal cortex where the dopamine release site density exceeds 1,000,000/mm^3, the release site density of the dorsal frontal cortex is 60,000/mm^3 (values from Table 2 in Descarries et al., 1987). Therefore, it does not seem appropriate to conclude that the dopamine release site density reported by Spühler and Hauri at 200,000/mm^3 is underestimated.

We thank the reviewer for this clarification. We have now removed the sentence mentioned by the reviewer.

The comparison of the effect of dopamine on the firing pattern recorded in the brain tissue (Gulledge and Jaffe, 1998) to the effect of the cAMP-modulation in the authors’ study is a reasonable approach to bridging the results between electrophysiological recording and theoretical studies. I think a quantitative description of the similarity between the two studies (ex, input resistance or the number of spikes before and after the neuromodulation) is more objective and reliable than the authors' description in this manuscript (“was in good agreement with…” in p.19).

We have replaced the phrase ”good agreement” by stating out that the numbers of spikes before and after neuromodulation were reproduced in the model as reported in Gulledge and Jaffe, 1998. The sentence mentioned by the reviewer (p. 6, not p.19) now reads:

The -10 mV shift applied in the Hay model as a model of cAMP-inhibiting neuromodulation was in agreement with the D2-mediated effects of 500 nM dopamine observed in [Gulledge and Jaffe, 1998]: our model reproduced the numbers of spikes (7 in control case, 5 under dopaminergic modulation, see Fig. 6 in [Gulledge and Jaffe, 1998]) in response to 1-second stimulation (Fig. S2E–F).
